# IFITM3 Restricts Influenza A Virus Entry by Blocking the Formation of Fusion Pores following Virus-Endosome Hemifusion

**DOI:** 10.1371/journal.ppat.1004048

**Published:** 2014-04-03

**Authors:** Tanay M. Desai, Mariana Marin, Christopher R. Chin, George Savidis, Abraham L. Brass, Gregory B. Melikyan

**Affiliations:** 1 Division of Pediatric Infectious Diseases, Emory University Children's Center, Atlanta, Georgia, United States of America; 2 Microbiology and Physiological Systems (MaPS) Department, University of Massachusetts Medical School, Worcester, Massachusetts, United States of America; 3 Children's Healthcare of Atlanta, Atlanta, Georgia, United States of America; Mount Sinai School of Medicine, United States of America

## Abstract

Interferon-induced transmembrane proteins (IFITMs) inhibit infection of diverse enveloped viruses, including the influenza A virus (IAV) which is thought to enter from late endosomes. Recent evidence suggests that IFITMs block virus hemifusion (lipid mixing in the absence of viral content release) by altering the properties of cell membranes. Consistent with this mechanism, excess cholesterol in late endosomes of IFITM-expressing cells has been reported to inhibit IAV entry. Here, we examined IAV restriction by IFITM3 protein using direct virus-cell fusion assay and single virus imaging in live cells. IFITM3 over-expression did not inhibit lipid mixing, but abrogated the release of viral content into the cytoplasm. Although late endosomes of IFITM3-expressing cells accumulated cholesterol, other interventions leading to aberrantly high levels of this lipid did not inhibit virus fusion. These results imply that excess cholesterol in late endosomes is not the mechanism by which IFITM3 inhibits the transition from hemifusion to full fusion. The IFITM3's ability to block fusion pore formation at a post-hemifusion stage shows that this protein stabilizes the cytoplasmic leaflet of endosomal membranes without adversely affecting the lumenal leaflet. We propose that IFITM3 interferes with pore formation either directly, through partitioning into the cytoplasmic leaflet of a hemifusion intermediate, or indirectly, by modulating the lipid/protein composition of this leaflet. Alternatively, IFITM3 may redirect IAV fusion to a non-productive pathway, perhaps by promoting fusion with intralumenal vesicles within multivesicular bodies/late endosomes.

## Author Summary

Interferon-induced transmembrane proteins (IFITMs) block infection of many enveloped viruses, including the influenza A virus (IAV) that enters from late endosomes. IFITMs are thought to prevent virus hemifusion (merger of contacting leaflets without formation of a fusion pore) by altering the properties of cell membranes. Here we performed single IAV imaging and found that IFITM3 did not interfere with hemifusion, but prevented complete fusion. Also, contrary to a current view that excess cholesterol in late endosomes of IFITM3-expressing cells inhibits IAV entry, we show that cholesterol-laden endosomes are permissive for virus fusion. The ability of IFITM3 to block the formation of fusion pores implies that this protein stabilizes the cytoplasmic leaflet of endosomal membranes, either directly or indirectly, through altering its physical properties. IFITM3 may also redirect IAV to a non-productive pathway by promoting fusion with intralumenal vesicles of late endosomes instead of their limiting membrane.

## Introduction

The recently identified interferon-induced transmembrane proteins (IFITMs) inhibit infection of diverse enveloped viruses [1]–[3]. Ectopic expression of IFITM1, -2 and -3 restricts a growing number of unrelated viruses, including IAV [1], [2], [4]–[7]. IFITM3 has been shown to potently restrict infection by IAV and the Respiratory Syncytial Virus *in vivo* [8]–[10]. In contrast, arenaviruses and some retroviruses, such as murine leukemia virus (MLV), are resistant to IFITM restriction [2], [6]. The IFITMs have been reported to inhibit HIV-1 entry, albeit less potently than IAV and apparently in a cell type-dependent manner [11]–[13].

The mechanism by which IFITMs inhibit infection of diverse viruses is not fully understood. IFITM2 and -3 are predominantly found in late endosomes (LE) and lysosomes [13], [14], whereas IFITM1 is also found at the cell periphery [4], [15]. Different membrane topologies of IFITMs have been proposed [16], but recent data suggests that IFITM3 is a type II transmembrane protein [17]. Accumulating evidence implies that IFITMs may interfere with virus-endosome fusion [1], [2], [5], [13], [14]. The fact that IFITMs seem to expand acidic intracellular compartments [13] indicates that the fusion block is downstream of the low pH trigger. Effective restriction of viruses that enter from the LE, such as IAV, Ebola virus (EBOV) and SARS coronavirus seems consistent with the cellular localization of IFITM2 and -3 proteins. However, these proteins also restrict Vesicular Stomatitis Virus (VSV) that appears to fuse with early endosomes [18].

IFITMs have been reported to curtail viral infection by modifying properties of cellular membranes, such as fluidity and spontaneous curvature [3], [5], [14]. These effects could be related, in part, to the accumulation of cholesterol in LE as a result of IFITM-mediated disruption of the interaction between the vesicle-membrane-protein-associated protein A (VAPA) and oxysterol-binding protein (OSBP) [14]. Since lipids play an important role in membrane fusion, these findings offer an attractive paradigm for a broad antiviral defense mechanism that involves altering the lipid composition of cellular membranes. The recent finding that amphotericin B, which forms complexes with sterols [19], rescues IAV infection in IFITM2- and IFITM3-expressing cells [20] is in line with the notion that cholesterol may be directly or indirectly involved in IAV restriction. However, lipid composition-based models do not readily explain the lack of restriction of amphotropic MLV and arenaviruses, which enter cells *via* distinct endocytic routes [21], [22]. These findings indicate that IFITMs may restrict virus entry from a subset of intracellular compartments. In order to define the mechanism of IFITM restriction, it is important to identify the viral entry step(s) targeted by these proteins, define compartments in which restriction occurs, and elucidate potential changes in intracellular membranes that may be responsible for this phenotype.

Here, we examined the mechanism of IFITM3 restriction of IAV using single particle imaging and a direct virus-cell fusion assay. Our results show that IFITM3 does not inhibit the lipid mixing stage of IAV fusion but blocks the release of viral contents into the cytosol, and that this phenotype does not correlate with cholesterol accumulation in intracellular compartments. Specifically, IFITM3 inhibits the conversion of hemifusion to fusion through a mechanism that does not rely on cholesterol accumulation. Together these findings reveal a previously unappreciated view of IFITM-mediated restriction and suggest new avenues of investigation to delineate the mechanism by which these proteins block infection.

## Results

### Virus- and cell type-dependent restriction of viral fusion by IFITM3

**Figure 1:**
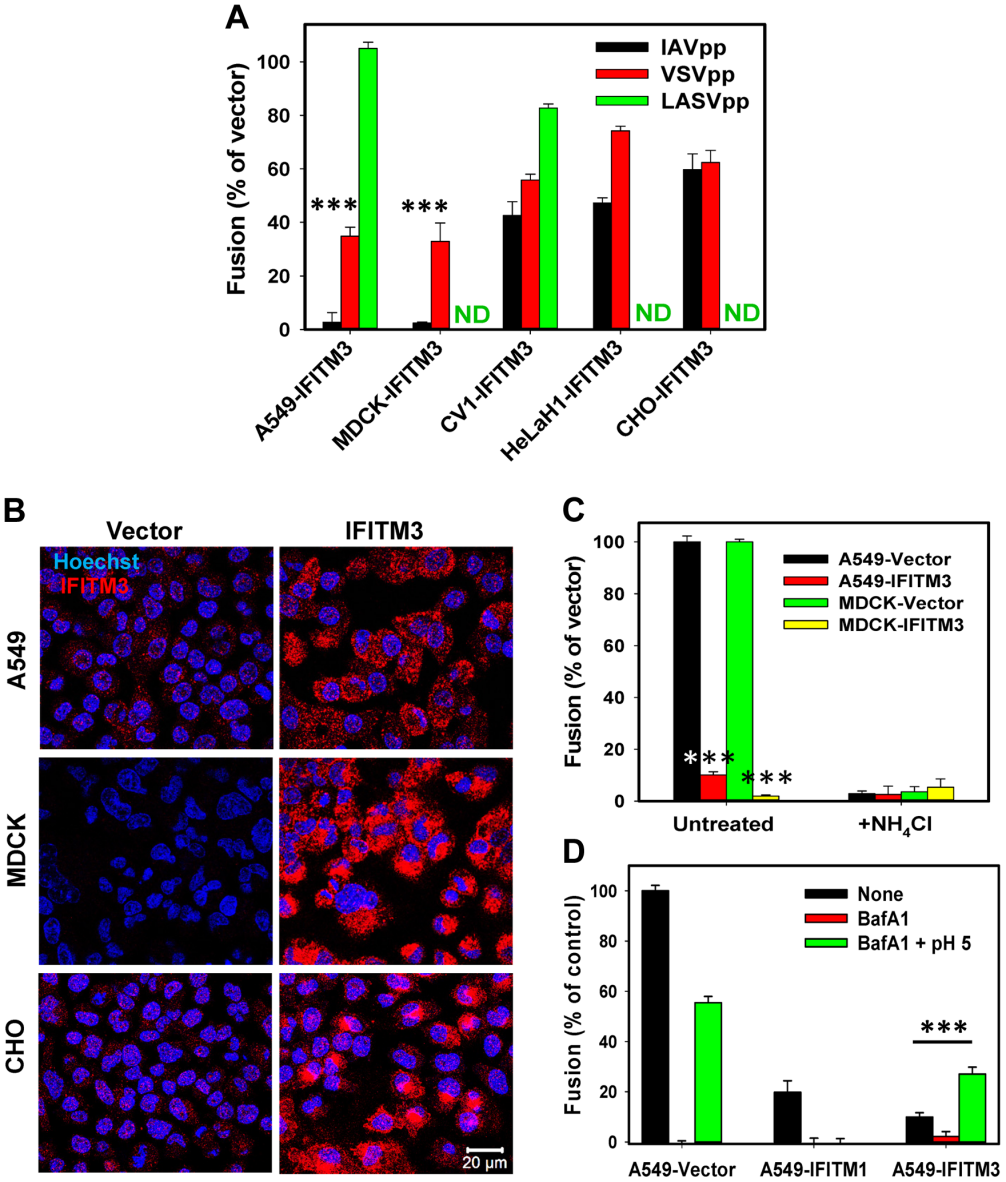
IFITM-mediated restriction of virus-endosome fusion in different cell types. (A) IFITM3-mediated inhibition of viral fusion with different cell types. BlaM-Vpr carrying pseudoviruses (IAVpp, VSVpp and LASVpp, MOI = 1) were bound to IFITM3- or vector-transduced A549, MDCK, CV1, HeLaH1 or CHO cells in the cold. Fusion was allowed to proceed for 90 min at 37°C and was measured by the BlaM assay, as described in Materials and Methods. ND, not determined. Data are means and SEM from 2 independent triplicate experiments. (B) IFITM3 expression patterns in A549, MDCK and CHO cells transduced with an empty vector (left) or IFITM3 (right). Cells were fixed, permeabilized and immunostained for IFITM3 (red), as described in Materials and Methods. The nuclear stain, Hoechst-3342, is shown in blue. (C) IFITM3 restricts fusion of influenza virus-like particles containing β-lactamase reporter protein fused to the influenza matrix protein-1 (BlaM1). Experiments were carried out as described above. Data are means and SEM from 2 independent triplicate experiments. (D) Exposure to low pH overcomes the IFITM3-mediated block of IAVpp fusion. To force pseudovirus fusion at the plasma membrane, A549 cells transduced with IFITM1, IFITM3 or an empty vector were pretreated with 50 nM BafA1 for 30 min at 37°C or left untreated. IAVpp/BlaM-Vpr pseudoviruses (MOI = 1) were bound to cells of in the cold and exposed to either a pre-warmed pH 5.0 MES-citrate buffer or neutral buffer for 10 min at 37°C and further incubated in growth medium (with or without BafA1) for 90 min at 37°C. Data are means and SEM from 2 independent triplicate experiments. ***, P<0.001 by two-tailed t-test.

We chose to focus on IFITM3 to study the mechanism of IAV restriction because this protein potently inhibits infection *in vitro* and *in vivo* [8]–[10]. Since published data suggest that IFITM3 likely inhibits the viral fusion step, a direct virus-cell fusion assay was employed to evaluate the extent of restriction in different cell lines [23]. HIV-1 particles carrying the β-lactamase-Vpr (BlaM-Vpr) chimera and pseudotyped with the influenza HA and NA proteins from the H1N1 A/WSN/33 strain (referred to as IAVpp) were allowed to fuse with cells transduced with an empty vector or with an IFITM3-expressing vector. The resulting cytosolic BlaM activity was measured as previously described [24]. Out of several cell lines tested, A549 and MDCK cells over-expressing IFITM3 were least permissive to IAVpp fusion (Fig. 1A). In agreement with the previous reports [2], [13], we found that IFITM3 over-expression partially inhibited VSV G glycoprotein-mediated fusion of pseudoviruses (VSVpp) carrying the BlaM-Vpr chimera (Fig. 1A). Similar to inhibition of IAVpp fusion, the IFITM3-mediated restriction of VSVpp was most potent in A549 and MDCK cells. As expected, fusion of particles pseudotyped with the Lassa fever virus glycoprotein (LASVpp), which directs virus entry through an IFITM3-resistant pathway [2], [6], was not considerably affected by IFITM3 over-expression.

We next checked if the strong suppression of virus fusion in A549 and MDCK cells was related to the level of IFITM3 expression. Immunostaining for IFITM3 in these and CHO cells which exhibited modest restriction of viral fusion (Fig. 1A) did not reveal a clear correlation between IFITM3 expression and inhibition of IAVpp or VSVpp fusion (Fig. 1B). Of note, potent IAV restriction in A549 and MDCK cells was not related to the usage of HIV-1 core-based pseudoviruses. Influenza virus-like particles containing the IAV BlaM-M1 chimera [25] also failed to efficiently fuse with A549-IFITM3 and MDCK-IFITM3 cells while fusing well with vector-transduced cells (Fig. 1C). We also found that both vector-transduced A549 and MDCK cells were highly susceptible to IAV infection, as determined by virus titration (see Materials and Methods). These two cell lines were therefore chosen for studies of IFITM3-mediated restriction described below.

IFITM-based restriction has been studied using a cell-cell fusion model, as well as by forcing viral fusion with the plasma membrane by lowering the pH [5], [20]. Since fusion with the plasma membrane is more amenable to mechanistic studies than endocytic entry, we asked whether IFITM3 can restrict forced IAV fusion. Exposure to acidic buffer induced IAVpp fusion with A549-Vector cells pretreated with Bafilomycin A1 (BafA1), which blocked low pH-dependent entry from endosomes (Fig. 1D). The extent of forced fusion was lower compared to the conventional entry route. By contrast, forced IAVpp fusion with A549-IFITM3 cells was ∼3-fold more efficient than endocytic fusion with cells not treated with low pH or BafA1, showing that IFITM3 does not restrict IAVpp fusion at the cell surface. Interestingly, IFITM1 suppressed IAVpp-plasma membrane fusion at low pH (Fig. 1D), in agreement with the Jaagsiekte sheep retrovirus (JSRV) and IAV fusion data [5], [20]. The inability of IFITM3 to block IAV fusion with the plasma membrane is consistent with its lower abundance at the cell surface [13], [14], [20] and shows that the mechanism of restriction must be studied in intracellular compartments.

### IFITM3 does not inhibit lipid mixing between IAV and acidic endosomes

Preponderance of evidence implies that hemifusion is a universal intermediate (reviewed in [26], [27]) that precedes the formation of a fusion pore. Having shown that IFITM3 over-expression inhibits viral fusion (Fig. 1A, C), we asked whether this protein also blocks the upstream hemifusion step. This was accomplished by labeling the A/PR/8/34 virus membrane with a self-quenching concentration of vybrant DiD (vDiD), using a modification of the previously published protocol [28]. Incorporation of self-quenching quantities of a lipophilic dye enables the visualization of single lipid mixing events based on the marked increase in fluorescence upon dye redistribution to an endosomal membrane (see for example [28], [29]).

Significantly, to control for fluctuations in the vDiD fluorescence caused by deviation from a focal plane, the viral surface proteins were labeled with the amine-reactive AlexaFluor-488 (AF488) dye. The relatively steady AF488 signal before and after hemifusion is allowed correcting for the vDiD intensity fluctuations due to moving in and out of focus. The vDiD/AF488 co-labeling protocol only modestly (<2-fold) reduced IAV infectivity compared to the mock-labeled viruses (Fig. S1A). Immunofluorescence staining of AF488-labeled virions with anti-HA antibodies revealed an excellent co-localization of the two signals (Fig. S1B, C), thus supporting the notion that AF488/vDiD-labeled particles are *bona fide* virions.

**Figure 2:**
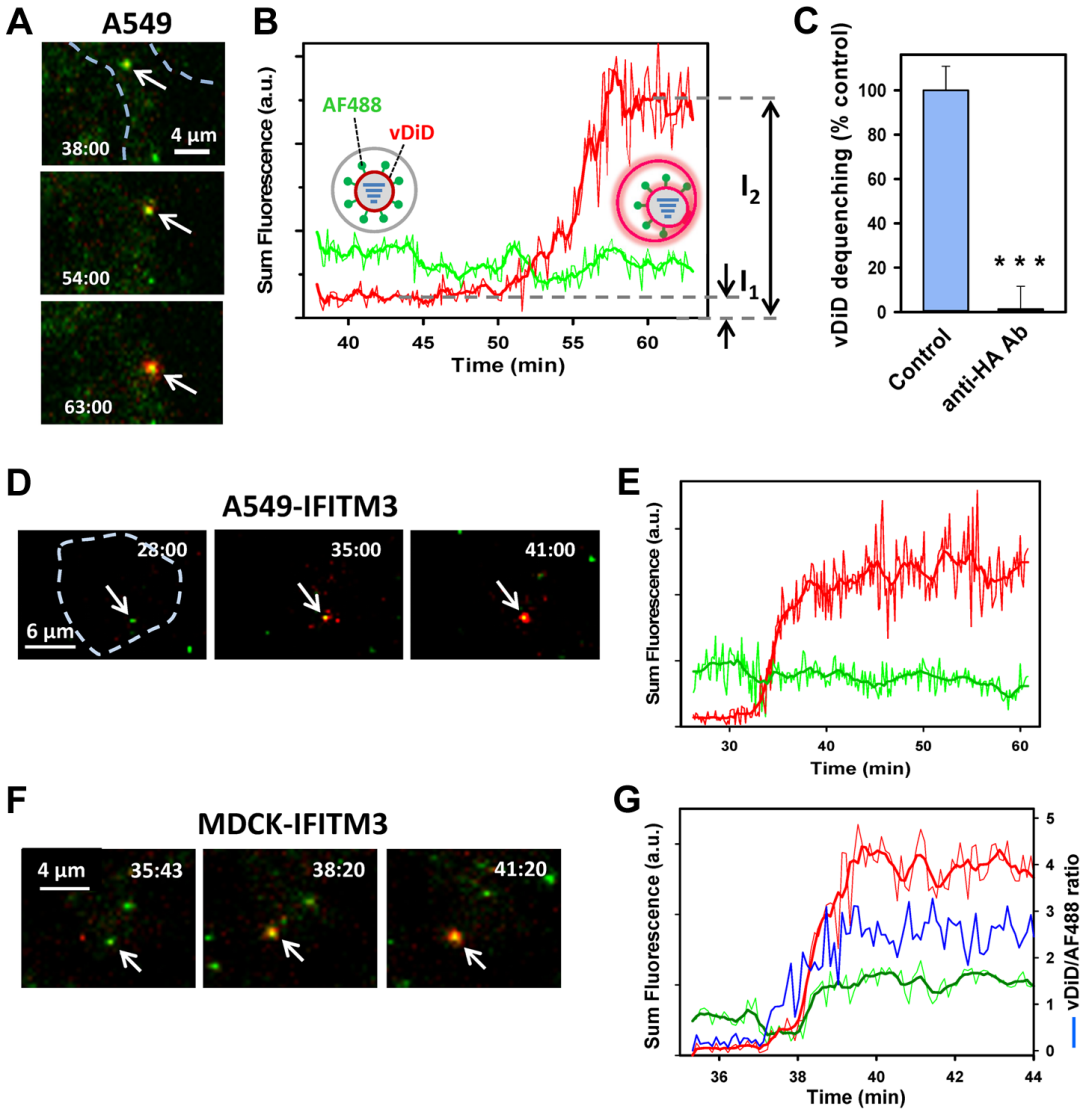
Lipid mixing between single IAV particles and endosomes in control and IFITM3-expressing cells. (A–E) IAV particles co-labeled with AF488 (green) and vDiD (red) were pre-bound to A549-Vector or A549-IFITM3 cells in the cold and incubated at 37°C for 1 h. Particles exchanging vDiD with endosomes (arrows in A and D) exhibited marked increase in red signal. (A, B) Images of vDiD dequenching (extended projections) and particle fluorescence intensities obtained by tracking virions in A549 cells. A schematic illustration of IAV hemifusion with an endosome (gray), which leads to vDiD dequenching, is overlaid on the graph. I_1_ and I_2_ are fluorescence intensities immediately before dequenching and at the peak of dequenching, respectively. (C) IAV lipid mixing activity in A549-Vector cells is blocked in the presence of anti-HA antibody. AF488- and vDiD-labeled IAV were pre-incubated with 20 µg/ml of polyclonal anti-IAV antibody (Millipore, Billerica, MA) for 1 h at room temperature. Viruses were then bound to A549-Vector cells in the cold by spinoculation, and entry was initiated with warm imaging buffer supplemented with 20 µg/ml of the antibody. Images were collected from 12 fields and the average fraction of AF488 particles with the vDiD signal above the threshold level was determined and normalized to control conditions without the antibody. ***, P<0.001. (D, E) Representative images and analysis of lipid mixing in A549-IFITM3 cells. (F, G) Representative images and analysis of lipid mixing in MDCK-IFITM3 cells. The ratio of vDiD and AF488 signals (blue line) shows robust increase in the red signal in spite of variations in the green channel caused by axial displacement of the virus. Thick lines were obtained by smoothing raw fluorescence intensity data (thin lines). Cell contours are shown by dashed lines in A and D. See also corresponding movies S1, S2 and S3.

**Figure 3:**
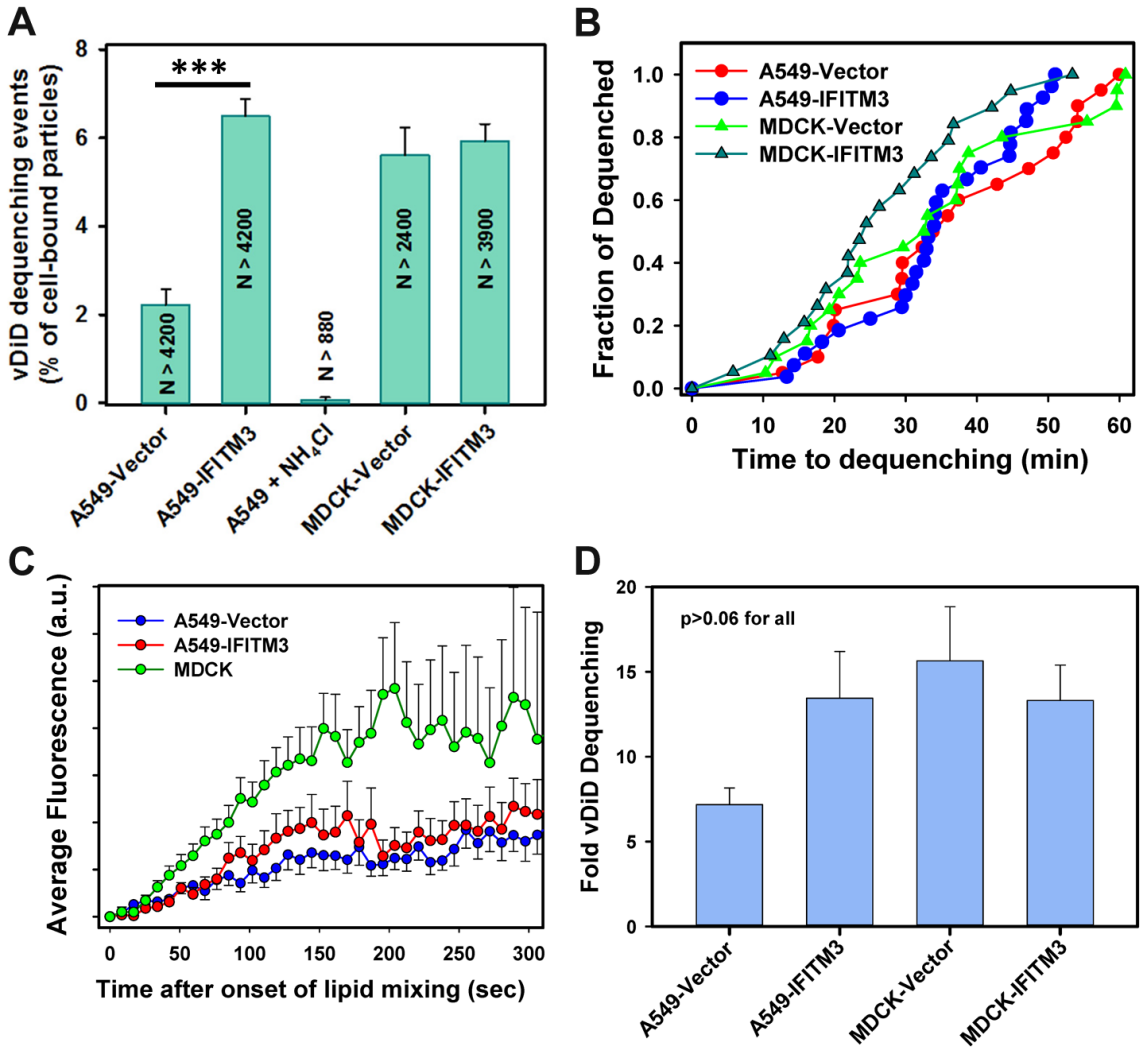
Analyses of the extent and kinetics of single IAV lipid mixing events. (A) The fraction of AF488-labeled particles undergoing lipid mixing in A549 transduced with an empty vector or IFITM3 and in MDCK cells. Control experiments in A549 cells were carried out in the presence of NH_4_Cl. Error bars are SEM from 11 independent experiments. ***, P<0.001. (B) The distribution of waiting times for onset of IAV lipid mixing in A549 and MDCK cells transduced with IFITM3 or an empty vector. The time intervals from shifting to 37°C to the onset of vDiD dequenching were determined, as described in Materials and Methods, and plotted as normalized fraction of events as a function of time. Pairwise comparison of all curves yields P>0.2. (C) Ensemble averages of initial vDiD dequenching profiles. The dequenching traces were aligned at the onset of hemifusion and averaged for each time point. Error bars are SEM. (D) The extent of vDiD dequenching was calculated based on I_2_/I_1_ ratio, as illustrated in Fig. 2B.

Labeled viruses were allowed to enter A549-Vector cells, and the resulting lipid mixing activity was examined by single particle tracking. A fraction of virions exhibited a marked increase in the vDiD signal (Fig. 2A, B). Redistribution of vDiD was mediated by low pH-dependent conformational changes in the IAV HA glycoprotein, as evidenced by potent inhibition of lipid mixing by anti-HA antibodies (Fig. 2C) and by NH_4_Cl (Fig. 3A). Without simultaneous monitoring of the viral content release into the cytoplasm, vDiD dequenching does not discriminate between hemifusion (operationally defined as lipid mixing without content transfer [30]) and full fusion. To avoid over-interpreting dequenching events, we will refer to these events as lipid mixing or hemifusion. A similar vDiD dequenching pattern was observed in MDCK cells transduced with an empty vector (data not shown). Analysis of lipid mixing showed that 2.2±0.4% and 5.6±0.6% of cell-bound particles released vDiD in A549 and MDCK cells, respectively (Fig. 3A). By comparison, a much greater fraction of virions (38.3±0.6%) hemifused with CHO cells (data not shown), in agreement with the previously reported data [28].

Importantly, IAV lipid mixing was readily detected in IFITM3^+^ A549 and MDCK cells (Figs. 2D–G and 3A). Not only was lipid mixing not inhibited in A549-IFITM3 cells, but a >3-fold greater fraction of particles released vDiD in these cells compared to control cells (Fig. 3A, P<0.001). By comparison, IFITM3 over-expression in MDCK cells did not significantly promote vDiD dequenching (Fig. 3A). Thus, contrary to the cell-cell fusion results [5], IFITM3 does not inhibit and can even promote IAV lipid mixing, consistent with the block of virus entry at a post-hemifusion stage. Accordingly, the addition of oleic acid, which augments hemifusion by altering spontaneous membrane curvature, did not rescue IAVpp or VSVpp fusion with A549-IFITM3 cells (Fig. S2). This is in agreement with the recent infectivity results [20], but in contrast with the rescue of fusion between JSRV Env- and IFITM-expressing cells by this fatty acid [5].

The higher frequency of vDiD dequenching in A549-IFITM3 cells could be caused by the increased endosome acidity compared to control cells [13]. However, the distribution of waiting times to the onset of lipid mixing was independent of IFITM3 expression or the type of target cells (A549 vs. MDCK, Fig. 3B, P = 0.37). The fact that the kinetic curves do not reach plateau indicates that IAV entry into A549 and MDCK cells is not completed within the first hour. Our results thus demonstrate that IFITM3 restricts the IAV fusion at a post-hemifusion step, most likely at the point of fusion pore opening, as evidenced by the dramatic decrease of the BlaM signal in A549 and MDCK cells expressing this protein (Fig. 1A).

### Lipid redistribution between IAV and endosomes is relatively slow and independent of IFITM3 expression

Under our conditions, vDiD dequenching was typically completed within a few minutes for both control and IFITM3^+^ cells (Fig. 2). This dequenching rate is much slower than sudden increases in fluorescence of the IAV membrane markers described previously [28], [31]. While a portion of vDiD dequenching could be completed within seconds (Fig. S3), these fast events were not common. Slow dequenching was also typical with the vDiD/AF488-labeled X31 virus, as well as with the X31 virus labeled with a 15-fold excess of DiD, using the published protocol for single virus imaging [28] (data not shown).

Slow vDiD dequenching during the first hour of virus-cell co-incubation did not appear to result from IAV degradation in LE/lysosomes, since the surface-exposed AF488 label persisted long after vDiD dequenching was completed and because anti-HA antibodies blocked vDiD dequenching (Fig. 2). In addition, we did not detect any correlation between the lag before the onset of lipid mixing and the vDiD dequenching slope (Fig. S4A). This result reinforces the notion that late lipid mixing events are mediated by HA and not by virus degradation. Control experiments, in which samples were not exposed to laser light during the first 30 min at 37°C, did not reveal fast dequenching events reaching completion in less than 1 min (data not shown). This control argues against phototoxicity-related attenuation of virus fusogenicity as the cause for sluggish lipid redistribution.

Since free vDiD diffusion between a virus and a small endosome should be completed in less than a second [32], [33], an initial membrane connection between IAV and an endosome must severely impair lipid movement. To assess whether early fusion intermediates in control and IFITM3^+^ cells restrict vDiD diffusion to the same extent, we examined the rate of vDiD dequenching. Single particle analysis revealed that, in A549 cells, the average vDiD dequenching profile (Fig. 3C) was independent of IFITM3 expression, as were the initial slopes of vDiD dequenching (Fig. S4B, P>0.5). These results indicate that IFITM3 over-expression does not affect the properties of fusion intermediates responsible for vDiD redistribution, such as the size and/or architecture of a hemifusion site (e.g., [34], [35]). We then asked whether the rate of vDiD dequenching varied depending on the cell type. The average rate of vDiD fluorescence increase in MDCK cells was ∼2-fold greater than in A549 cells (Figs. 3C and S4B, P<0.02). This demonstrates our ability to detect changes in the rate of vDiD transfer and shows that lipid transfer lasts several minutes irrespective of the cell type.

We also examined the final extent of vDiD dequenching, which is proportional to the surface area of a target membrane over which it redistributes. This parameter was not significantly affected by IFITM3 expression in A549 cells or by the cell type (MDCK vs. A549 cells, Fig. 3D). Together, similar kinetics and extents of viral lipid dilution in control and IFITM3^+^ cells suggest that neither the size/architecture of early fusion intermediates nor the surface area of target endosomes is considerably affected by IFITM3 expression.

To investigate the relationship between lipid mixing and productive IAV infection, we compared the fraction of cells “receiving” at least one vDiD dequenching event in live cell imaging experiments to the fraction of cells that got infected under the same conditions. The only difference was that virus imaging was not continued beyond 1 h after initiation of fusion, whereas infection proceeded overnight. We found that one or more vDiD dequenching events occurred in 15% of A549 cells while 44% of cells got infected (Fig. S5). Under the same conditions, 20% of MDCK cells “hosted” one or more dequenching events and 36% were infected. The greater fraction of infected cells compared to those permissive to hemifusion is likely due to the shorter time widow for single virus imaging, which is likely to miss late vDiD dequencing events (Fig. 3B). The lower apparent fraction of cells supporting vDiD dequenching could also be caused by the presence of viruses that did not incorporate self-quenching amounts of vDiD. Importantly, the comparable efficiencies of lipid mixing and infection, indicate that the former events likely culminate in productive infection.

### IFITM3 inhibits the formation of small fusion pores

**Figure 4:**
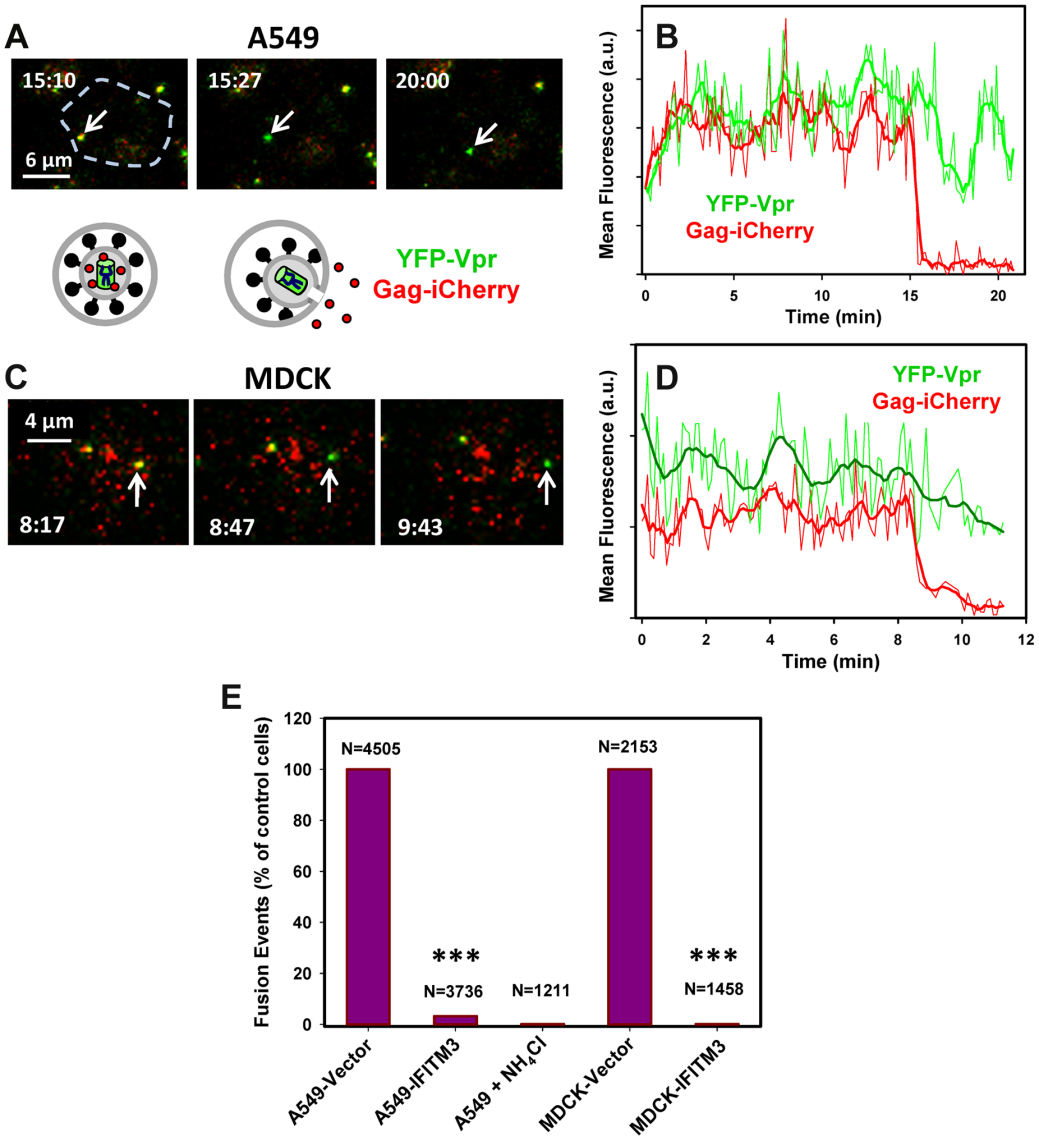
IFITM3 blocks fusion pore formation between single influenza viruses and endosomes. Pseudoviruses bearing WSN HA and NA glycoproteins were co-labeled with HIV-1 Gag-iCherry (viral content marker, red) and YFP-Vpr (viral core marker, green). Viruses were pre-bound in the cold to A549-Vector cells (A, B) or MDCK-Vector cells (C, D) and their entry was initiated by raising the temperature. (A, C) Images of IAVpp are extended projections of 3 Z-stacks illustrating the loss of the mCherry signal (arrow) upon virus-endosome fusion. A schematic illustration between image panels A and C illustrates fusion between the YFP-Vpr (green) and Gag-iCherry (red) labeled IAVpp and an endosome (gray). (B, D) Mean mCherry and YFP fluorescence intensities obtained by tracking the particles shown in panels A and C. (E) Normalized efficiencies of IAVpp fusion (content release) with A549 and MDCK cells transduced with an empty vector or with IFITM3. The middle bar shows the lack of mCherry release in A549-Vector cells in the presence of NH_4_Cl. ***, P<0.001. See movies S4 and S5.

To determine whether IFITM3 impairs the IAV's ability to form small fusion pores, we attempted to load the virus with a content marker by soaking in a concentrated solution of sulforhodamine B, as described in [36]. However, only a small fraction of AF488-labeled particles stained with sulforhodamine, and the retained dye was lost in live cell experiments under conditions that blocked IAV fusion (data not shown). We therefore resorted to using HIV pseudoviruses bearing A/WSN/33 HA and NA glycoproteins and co-labeled with the capsid marker, YFP-Vpr, and the content marker, Gag-iCherry [24], [37]. Upon virus maturation, the “internal” mCherry is proteolytically cleaved off the HIV-1 Gag-iCherry and released through a fusion pore, as manifested by the loss of the red signal (Fig. 4 and [37]). The YFP-Vpr signal, which remained associated with the viral core after fusion, provided a reference signal for single particle tracking.

Under our conditions ∼1% of double-labeled pseudoviruses entering A549-Vector cells lost their content marker, while approximately 2% fused with MDCK-Vector cells. In sharp contrast, the mCherry release in IFITM3^+^ A549 and MDCK cells or in vector-transduced cells in the presence of NH_4_Cl could not be detected (Fig. 4E, P<0.001). Thus, IFITM3 does not adversely affect IAV hemifusion but severely inhibits viral content release into the cytoplasm. Together these findings suggest that the mechanism of IFITM3-mediated restriction arises from the entrapment of viruses at a hemifusion intermediate prior to fusion pore formation.

### Cholesterol accumulation in endosomes does not inhibit viral fusion

**Figure 5:**
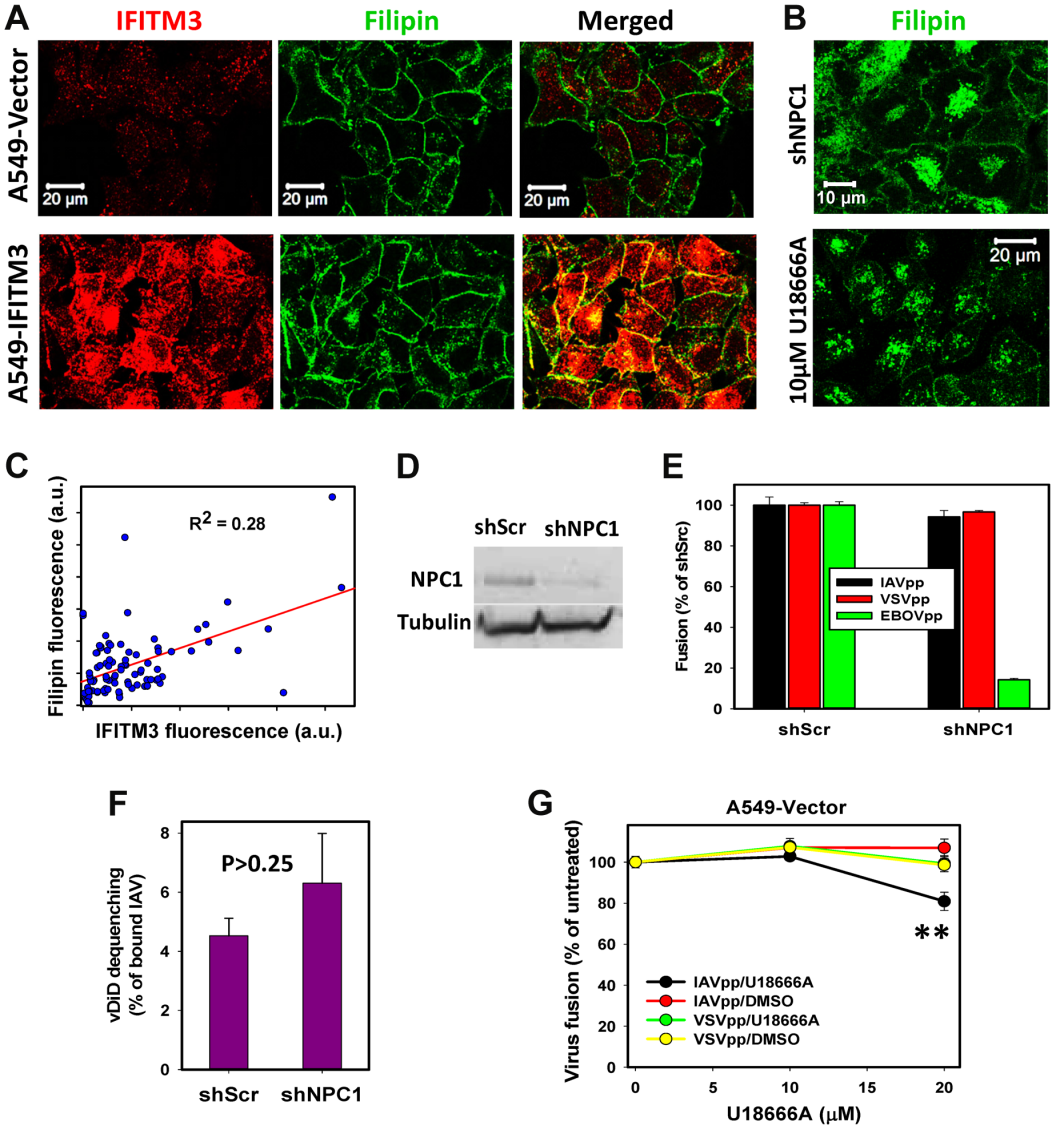
IFITM3 restriction of IAV fusion with A549 cells is not related to cholesterol accumulation in endosomes. (A) Sub-cellular distributions of cholesterol and IFITM3 in A549-Vector and A549-IFITM3 cells. Cholesterol and IFITM3 staining was done using filipin and anti-IFITM3 antibody, respectively. Images show confocal slices through the middle section of cells. (B) Filipin staining of A549 cells transduced with shRNA against NPC1 (upper panel) and of cells pretreated with 10 µM U18666A for 18 h (lower panel). (C) Intracellular filipin and IFITM3 signals are poorly correlated. Individual regions of interests within 91 cells were drawn to exclude plasma membrane fluorescence, followed by background subtraction and summation of fluorescence intensity within each region of interest. (D) Western blotting analysis of NPC1 expression in A549 cells transduced with scrambled shRNA (A549.shScr) or with shRNA specific to NPC1 (A549.shNPC1). Tubulin was used as a loading control. (E) IAVpp, VSVpp and EBOVpp fusion with A549.shScr and A549.shNPC1 cells measured by the BlaM assay. Data are means and SEM from 2 triplicate experiments (IAVpp and VSVpp) and 1 triplicate experiment (EBOVpp). (F) Single IAV lipid mixing activity in A549.shScr and A549.shNPC1 cells. Cells were allowed to bind AF488- and vDiD-labeled IAV in the cold and incubated at 37°C for 1 h. The number of vDiD dequenching events was normalized to the total number of cell-bound particles from two experiments (n>690 particles) for each cell line. Error bars are standard deviations. (G) Dose-dependence of U18666A effect on viral fusion. A549 cells were pre-incubated for 18 h with indicated concentrations of U18666A or DMSO (control). BlaM-Vpr-carrying pseudoviruses (MOI = 1) were allowed to fuse with cells for 90 min at 37°C in the presence of U18666A or DMSO. Data are means and SEM from 2 triplicate experiments. **, P = 0.005.

A recent study has shown that, through disrupting the interaction between VAPA and OSBP, IFITM3 causes cholesterol accumulation in LE [14]. Based on this finding, the authors proposed that high levels of endosomal cholesterol may inhibit IAV fusion and/or the release of nucleocapsid. Staining with filipin revealed that IFITM3^+^ A549 cells exhibited increased levels of intracellular cholesterol (Fig. 5A). However, the filipin signal was still primarily associated with the plasma membrane and the total cellular cholesterol was not elevated in IFITM3^+^ cells (Fig. S6). In addition, the overall intensity of intracellular cholesterol poorly correlated with the level of IFITM3 expression (Fig. 5C).

By comparison, pretreatment of A549-Vector cells with U18666A, which inhibits transport of LDL-derived cholesterol from LE/lysosomes (reviewed in [38]), resulted in a dramatic shift in the filipin staining pattern from the plasma membrane to endosomes (Fig. 5B). Aberrant accumulation of cholesterol in LE is also known to occur in cells lacking the functional NPC1 cholesterol transporter [39]. We therefore knocked down NPC1 expression in A549 cells using shRNA (shNPC1, Fig. 5D) and examined the resulting cholesterol distribution (Fig. 5B). Reduced NPC1 expression correlated with excess cholesterol in intracellular compartments, which was also much more pronounced than endosomal filipin staining in A549-IFITM3 cells.

We next asked whether the cholesterol accumulation induced by U18666A pretreatment or by down regulation of NPC1 can phenocopy the IFITM3-mediated restriction of viral fusion. Neither IAV lipid mixing (vDiD dequenching) nor fusion (BlaM signal) was inhibited by silencing NPC1 in A549 cells (Fig. 5E, F). VSVpp also fused with shNPC1-transduced cells as efficiently as with control cells (Fig. 5E). These results show that excess cholesterol does not inhibit viral fusion or hemifusion. In control experiments, silencing the NPC1 expression potently suppressed fusion of Ebola GP-pseudotyped particles (EBOVpp, Fig. 5E), which use NPC1 as a receptor [40], [41]. Similar to the NPC1 knockdown phenotype, pretreatment of A549 cells with 10 µM U18666A, which caused cholesterol buildup in endosomes (Fig. 5B), did not inhibit fusion of IAVpp or VSVpp (Fig. 5G). As will be shown below for MDCK cells, higher doses of U18666A can inhibit viral fusion (Fig. 5G), but this effect is due to elevation of endosomal pH as opposed to cholesterol accumulation in endosomes.

**Figure 6:**
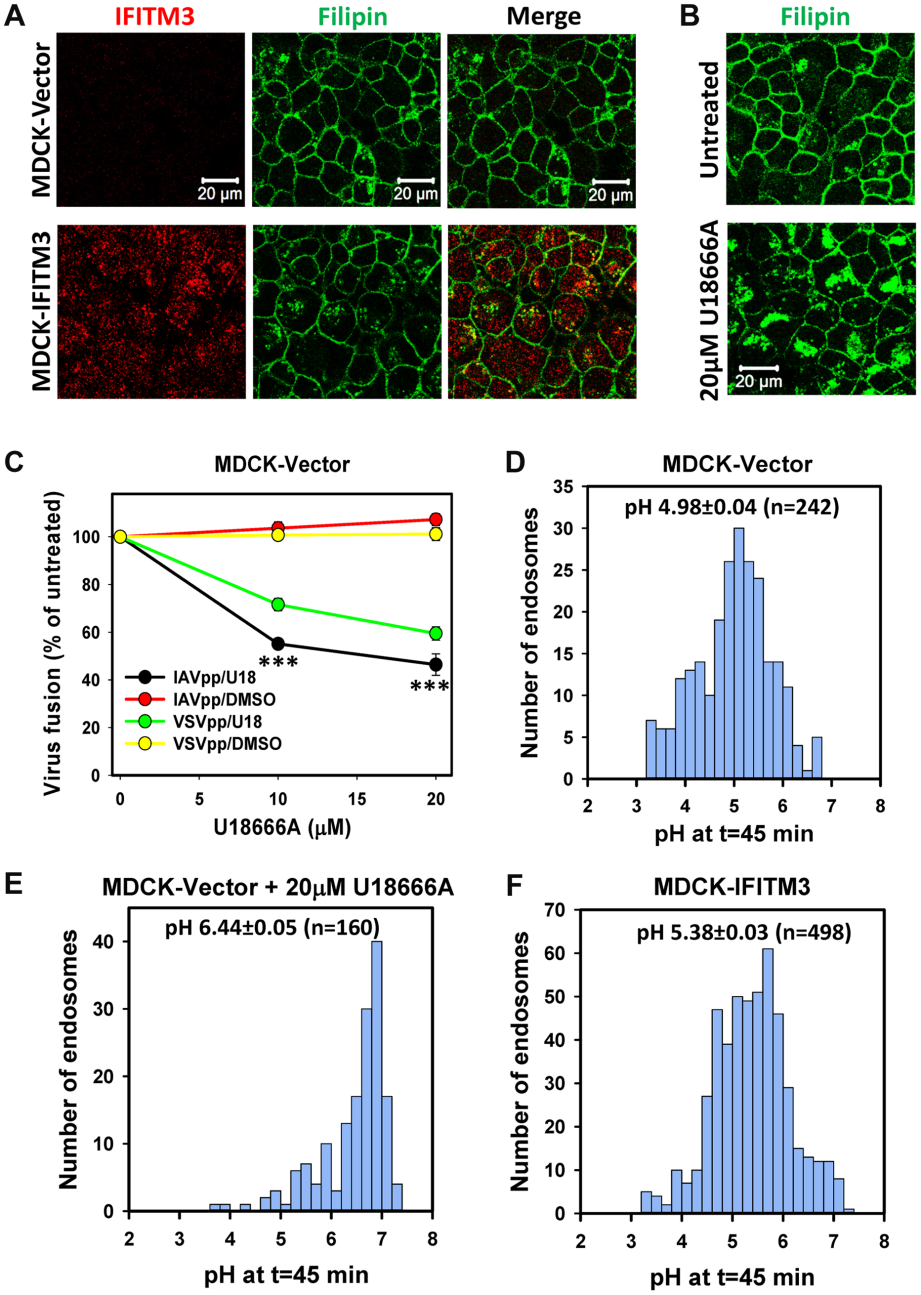
IFITM3-mediated restriction of IAV fusion is not related to cholesterol accumulation in endosomes of MDCK cells. (A) Sub-cellular distributions of cholesterol (filipin staining) and IFITM3 (antibody staining) in MDCK-Vector and MDCK-IFITM3 cells. Images show confocal sections through the middle of cells. (B) Filipin staining of MDCK cells pretreated with 20 µM U18666A for 18 h or mock-treated cells. (C) Dose-dependence of U18666A effect on viral fusion. MDCK-Vector cells were pretreated for 18 h with indicated concentrations of U18666A or DMSO (control). BlaM-Vpr-carrying pseudoviruses (MOI = 1) were allowed to fuse with cells for 90 min at 37°C in the presence of U18666A or DMSO. Data are means and SEM from 2 triplicate experiments. (D–F) pH distributions in IAV-carrying endosomes of MDCK cells measured using AF488- and CypHer5E-labeled viruses. Viruses were pre-bound to cells in the cold and incubated at 37°C for 45 min before acquiring images. Calculated pH values are shown for MDCK cells without (D) and with pretreatment with 20 µM U18666A for 18 h (E), as well as for MDCK-IFITM3 cells (F). Data are from 10 image fields each.

To generalize the effects of excess cholesterol in A549 cells, we tested whether endosomal cholesterol can inhibit viral fusion in MDCK cells. As in A549 cells, IFITM3 over-expression in MDCK cells caused moderate accumulation of cholesterol in endosomes (Fig. 6A), while pre-treatment with U18666A caused a much more dramatic buildup of intracellular cholesterol (Fig. 6B). However, unlike A549 cells, IAVpp and VSVpp fusion was significantly inhibited in U18666A-treated MDCK cells (Fig. 6C). Since prolonged exposure to U18666A has been reported to raise endosomal pH [42], we sought to determine if insufficiently acidic pH could prevent IAV hemifusion/fusion with pretreated MDCK cells.

The pH in IAV-carrying endosomes was measured using virions co-labeled with the pH-insensitive AF488 (green) and CypHer5E (red), which fluoresces brighter at acidic pH [28] (Fig. S7A). Cells were incubated with viruses for 45 min, and the red/green signal ratio from individual particles was measured (Fig. S7B). The average pH in virus-containing endosomes of MDCK-IFITM3 cells was slightly less acidic than in control cells: 5.38±0.03 (n = 498) vs. 4.98±0.04 (n = 242), respectively (Fig. 6D and F, P<0.001). Interestingly, as shown in Figure 6E, endosomal pH in U18666A-treated MDCK cells was markedly shifted to neutral values (6.44±0.05, n = 160, P<0.001). Since the pH threshold for triggering A/PR/8/34 fusion is reported to be around 5.6 [43], elevation of endosomal pH in U18666A-treated MDCK cells is the likely cause of inhibition of viral fusion. Together our results imply that U18666A most likely attenuates IAV fusion with MDCK cells by raising endosomal pH and not through inducing cholesterol accumulation.

**Figure 7:**
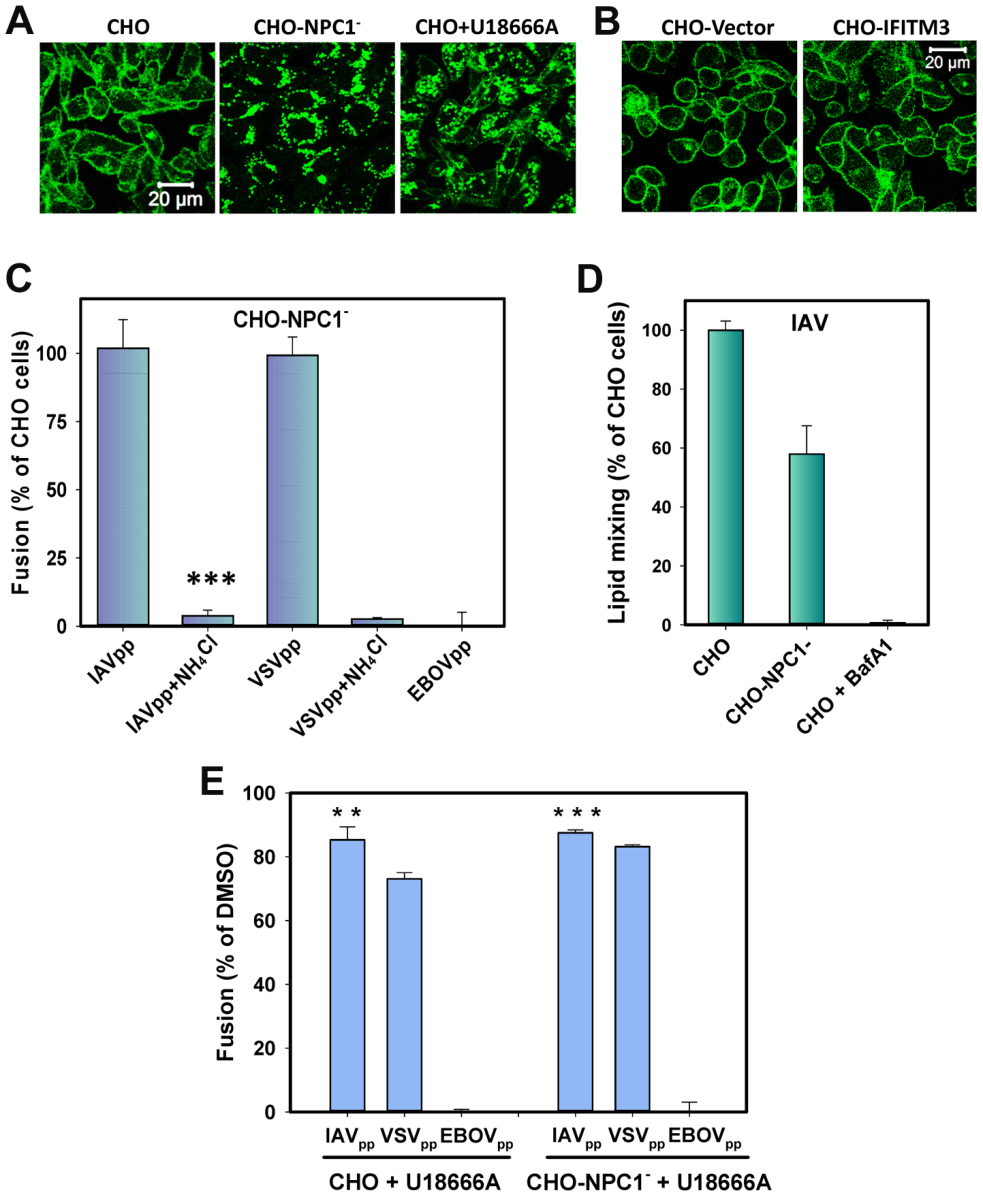
Cholesterol accumulation in endosomes of CHO cells does not inhibit viral fusion. (A) Filipin staining of untreated and U18666A-treated (40 µM) CHO cells and of CHO-NPC1^−^ cells devoid of NPC1. (B) Filipin staining of CHO-Vector and CHO-IFITM3 cells. Images in panels A and B show confocal sections through the middle of cells. (B) Confocal images of CHO-Vector and CHO-IFITM3 cells stained with filipin. (C) IAVpp (MOI = 2), VSVpp (MOI = 1) or EBOVpp (MOI = 2) were pre-bound to CHO or CHO-NPC1^−^ cells in the cold, incubated at 37°C for 90 min, and the resulting fusion activity was measured by the BlaM assay. Results are plotted as the relative extents of fusion CHO-NPC1^−^ cells after normalizing to fusion with CHO cells. Control experiments were carried out in 70 mM NH_4_Cl. Data are means and SEM from 3 triplicate experiments. (D) The frequency of lipid mixing in CHO (n = 576) and CHO-NPC1^−^ cells (n = 1241). Pre-treatment with 0.2 µM BafA1 for 30 min followed by initiation with imaging buffer containing BafA1and 70 mM NH_4_Cl inhibited the fusion activity: only 4 out of 1532 particles underwent lipid mixing. Error bars are standard deviations from at least 4 experiments. (E) Pretreatment of CHO cells with U18666A (40 µM, 8 h) modestly diminishes IAVpp or VSVpp fusion and abrogates EBOVpp fusion, as measured by the BlaM assay. Data are means and SEM from 2 triplicate experiments. ***, P<0.001; **, P<0.02.

We also took advantage of the available CHO cell line that does not express NPC1 [44] to further ascertain the role of endosomal cholesterol in IAV fusion. These cells (designated CHO-NPC1^−^) exhibited exaggerated endosomal cholesterol staining, in sharp contrast to a peripheral staining pattern in parental CHO cells (Fig. 7A). In spite of the high endosomal cholesterol content in CHO-NPC1^−^ cells and of the elevated level of total cholesterol (Fig. S6), IAVpp fused with these cells as efficiently as with parental cells (Fig. 7C). The NPC1-null cells also supported IAV lipid mixing, albeit at somewhat reduced level compared to control (Figs. 7D and S8). Pretreatment of CHO cells with U18666A also trapped cholesterol in endosomes and raised the total cholesterol content (Figs. 7B and S6), but only modestly diminished the extent of IAVpp or VSVpp fusion (Fig. 7E). Interestingly, in contrast to the decreased endosome acidity in MDCK cells, endosomes in U18666A-treated CHO cells were more acidic than in control cells (Fig. S9). In control experiments, both the lack of NPC1 expression and U18666A pretreatment blocked EBOVpp fusion (Fig. 7C, E), consistent with its reliance on NPC1 receptor and high sensitivity to disruptions of cholesterol transport [45].

Together, our results show that the cholesterol accumulation achieved through two different interventions – U18666A pretreatment and NPC1 silencing – does not phenocopy IFITM3-mediated restriction of viral fusion. This implies that (i) elevated levels of endosomal cholesterol do not generally confer resistance to viral fusion, and (ii) the mechanism by which IFITM3 blocks transition from hemifusion to full fusion is not through the mislocalization of cholesterol.

## Discussion

**Figure 8:**
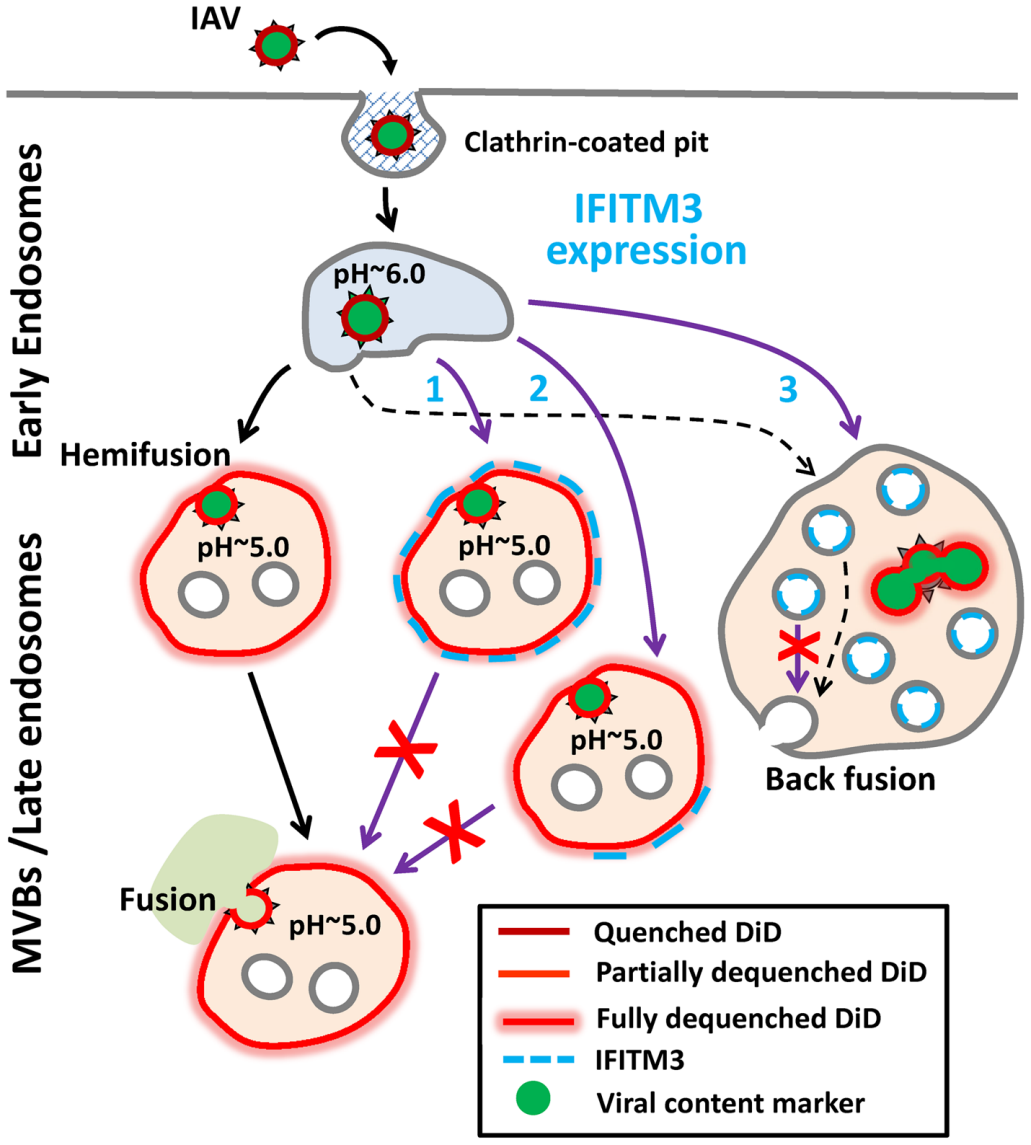
Models for IFITM3-mediated restriction of IAV infection. Purple arrows illustrate possible mechanisms of the IAV restriction by IFITM3: direct (Pathway 1) and indirect (Pathway 2) inhibition of transition from hemifusion to full fusion at the limiting membrane of an endosomes, as well as non-productive IAV fusion with ILVs in the absence of back fusion (Pathway 3). Partial dilution/dequenching of viral vDiD upon hemifusion/fusion is shown by lighter red color and full dequenching is shown by light red glow. Alternative endosomal localizations of IFITM3 (limiting membrane vs. ILVs) are shown. Dashed black arrow illustrates possible IAV fusion pathway in cells expressing low, endogenous levels of IFITM3.

The IFITMs restrict the cellular entry of multiple pathogenic enveloped viruses. Recent studies lead to a model that IFITMs inhibit virus-host hemifusion [5] and that the membrane-rigidifying properties of cholesterol may contribute to antiviral actions [14]. In contrast to these studies, our results now demonstrate that IFITM3 prevents the release of viral genomes into the cytosol by inhibiting viral entry after hemifusion but prior to fusion pore formation (Fig. 8). Moreover, we found that IFITM3 can promote hemifusion in some cells, perhaps secondary to its acidifying the endosomal pathway. IFITM3 therefore does not negatively regulate the properties of contacting leaflets involved in hemifusion, but stabilizes the cytoplasmic leaflet of the endosomal membrane, thereby disfavoring the formation of fusion pores [35]. In one potential scenario IFITM3 is located directly at the site of arrested hemifusion, perhaps “toughening” the endosomal membrane to create a barrier to viral entry (Pathway 1). A considerable colocalization of IFITM3 with internalized IAV ([3] and Fig. S10) is consistent with Pathway 1's direct mechanism of inhibition. Alternatively, IFITM3 might arrest hemifusion through an indirect mechanism, perhaps involving modulation of lipid and/or protein composition of the cytoplasmic leaflet (Pathway 2). Recent findings that changes in global membrane properties interfere with productive entry would appear to support an indirect mechanism [5], [14].

Lipids, such as unsaturated fatty acids and cholesterol that confer negative spontaneous curvature to membranes can promote hemifusion (a net negative curvature structure) and disfavor a fusion pore (a net positive curvature intermediate), as has been previously shown for oleic acid [35]. Although this prediction is consistent with efficient lipid mixing in endosomes of IFITM3^+^ cells observed in our imaging experiments, several studies [20], [46]–[48] and our own results do not support cholesterol accumulation as playing a role in fusion inhibition. We found that cholesterol-laden endosomes in cells pretreated with U18666A or expressing undetectable/low levels of NPC1 supported efficient viral fusion. It is thus possible that IFITM3 interferes with cellular functions of VAPA other than the interaction with OSBP, such as regulation of SNAREs and modulation of lateral mobility of membrane proteins (reviewed in [49]).

IFITM3 appears to induce the formation multivesicular bodies and increase the number of ILVs [13], [14]. One can therefore envision that IFITM3 may inhibit infection by redirecting viruses to a non-productive pathway, perhaps involving fusion with ILVs instead of the limiting membrane of LE (Fig. 8, Pathway 3). If, as suggested in [14], IFITM3 disallows back fusion of ILVs with the limiting membrane, then virus-ILV fusion products will likely be degraded. Indeed, back fusion has been implicated in the VSV core release into the cytosol following the virus-ILV fusion [50]. It should be stressed that this “fusion decoy” model does not explain the ability of IFITM1 to interfere with fusion at the cell surface ([5] and Fig. 1D). It is also not clear why the Old World arenaviruses, which have been reported to enter from MVBs [51], are not restricted by IFITMs.

The indistinguishable extents of vDiD dequenching in control and IFITM3^+^ cells (Fig. 3D) indicate that target endosomes have similar sizes. While this appears to argue against redirection of IAV fusion to small ILVs, the lack of a post-hemifusion decay of vDiD fluorescence in A549 and MDCK cells (Figs. 2 and S3) is consistent with IAV fusion with abundant ILVs in endosomes of IFITM3^+^ cells. This is because a lipophilic dye in the limiting membrane of an endosome should be quickly removed through membrane trafficking [24], [31], [52]. Because post-dequenching decay was not observed irrespective of the level of IFITM3 expression, it is possible that IAV may infect several cell lines by fusing with small intralumenal vesicles followed by the nucleocapsid release through back fusion (Fig. 8, dashed black arrows). This pathway could explain the similar extents and rates of vDiD dequenching in control and IFITM3-expressing cells, which are indicative of similar lipid intermediates and of the size of a target membrane, respectively.

As discussed above, slow vDiD dequenching observed by single IAV imaging can be rationalized in the context of fusion with the limiting membrane of endosomes (Pathways 1 and 2), as well as in the context of fusion with ILVs (Pathway 3). Slow dilution of this dye in Pathway 3 could occur through multiple rounds of IAV fusion with small ILVs, whereas Pathways 1 and 2 would predict restricted lipid diffusion through early fusion intermediates formed at the limiting membrane. Although the latter notion is in agreement with the reported restriction of lipid movement through hemifusion sites and small fusion pores [34], [35], [53], [54], these intermediates are usually short-lived under physiological conditions and tend to resolve into larger structures that do not impair lipid movement [28], [32], [35]. Clearly, more detailed studies of virus-endosome hemifusion and fusion are needed to understand the nature of slow lipid redistribution between IAV and endosomes.

The IFITMs may now arguably be one of the most broadly acting and clinically relevant restriction factor families [1], [3]. While both IFITM3's membrane-associated topology and its localization to the site of viral attenuation suggest it acts to restrict viral entry *via* a direct mechanism, additional work remains to be done to fully elucidate its actions. Nonetheless, as the primary effector of IFN's anti-IAV actions, IFITM3 represents a previously unappreciated class of restriction factor that prevents viral entry by stabilizing a hemifusion intermediate, likely comprised of an invading virus fatally tethered to the interior of the endosome's limiting membrane. Future single virus experiments combining the detection of both viral lipid and content release events (see for example [52]) should provide further insights into IAV entry pathways and the mechanism of IFITM3-mediated restriction. Indeed, such efforts may also bring to light unknown viral countermeasures, which are perhaps employed by the IFITM-resistant New and Old World arenaviruses.

## Materials and Methods

### Cell lines, plasmids and reagents

HEK 293T/17 cells and human lung epithelial A549 cells were obtained from ATCC (Manassas, VA) and grown as previously described [55]. Wild-type CHO cells and CHO-NPC1^−^ cells, a gift from Dr. L. Liscum (Tufts University) [44], were grown in Alpha-MEM (Quality Biological Inc, Gaithersburg, MD) supplemented with 10% FBS and penicillin-streptomycin. The A549, MDCK, HeLaH1 and CHO cells stably expressing IFITM3 or IFITM1 were obtained by transducing with VSV-G-pseudotyped viruses encoding wild-type IFITM3 and IFITM1 or with the vector pQCXIP (Clontech) and selecting with puromycin, as described previously [2].

The pR8ΔEnv, BlaM-Vpr, pcRev, HIV-1 Gag-iCherryΔEnv and pMDG VSV G expression vectors were described previously [37], [55]. The YFP-Vpr was a gift from Dr. T. Hope (Northwestern University). The pCAGGS vectors encoding influenza H1N1 WSN HA and NA were provided by Donna Tscerne and Peter Palese, and the pCAGGS BlaM1 (WSN) plasmid was a gift from Dr. A. Garcia-Sastre (Mount Sinai). Vectors expressing phCMV-GPc Lassa and pcDNA3.1-Ebola GP (Zaire) were gifts from Dr. F.-L. Cosset (Université de Lyon, France) [56] and Dr. L. Rong (University of Illinois) [57], respectively.

U18666A was from Tocris Bioscience (Bristol, UK). Poly-L-lysine, filipin, sulphorhodamine B Bafilomycin A1 and the Cholesterol Kit were from Sigma-Aldrich. AlexaFluor-488 amine-reactive carboxylic acid, vybrant-DiD (vDiD, 1,1′-dioctadecyl-3,3,3′,3′-tetramethylindodicarbocyanine,4-chlorobenzenesulfonate salt), Hoechst-33342 and Live Cell Imaging buffer were purchased from Life Technologies (Grand Island, NY). CypHer5E Mono NHS Ester was from GE Healthcare (Pittsburgh, PA). Antibodies used were rabbit anti-IFITM3 (to N-terminus) from Abgent (San Diego, CA), mouse anti-IAV-NP and goat anti-IAV-polyclonal antibodies from Millipore (Billerica, MA), rat anti-mouse-IgG-FITC from eBioscience (San Diego, CA), and goat anti-rabbit-Cy5 from Jackson Immunoresearch (West Grove, PA).

### Pseudovirus production, labeling and characterization

Pseudovirus production and titration were described previously [58]. Pseudoviruses were produced by transfecting HEK293T/17 cells using JetPRIME transfection reagent (Polyplus-transfection SA, NY). For LASV and EBOV pseudoviruses, 5 µg of the phCMV-GPc Lassa or 5 µg of the pcDNA3.1-Ebola GP was included in the transfection mixture. Fluorescently labeled influenza pseudoviruses were produced using 1 µg of pR8ΔEnv, 2 µg of HIV-1 Gag-iCherryΔEnv [37], 2 µg of YFP-Vpr, 1 µg of pcRev, and 2 µg of each WSN HA- and NA-expressing vectors. Ebola GP pseudoviruses were concentrated 10×, using Lenti-X™ Concentrator (Clontech, Mountain View, CA). To generate influenza BlaM1 VLPs, HEK293T cells were transfected with pCAGGS-BlaM1 (5 µg) and 2.5 µg of each pCAGGS-WSN HA and pCAGGS-WSN NA. After 12 h, the transfection reagent was removed, and cells were further cultivated in phenol red-free growth medium.

The influenza virus surface proteins and the lipid membrane were labeled with AF488 and vDiD, respectively. A hundred µg of influenza virus from the purified H1N1 A/PR/8/34 stock (2 mg/ml, Charles River, CT) was diluted in 95 µl of sodium bicarbonate buffer (pH 9.0) supplemented with 50 µM AF488. The mixture was incubated for 30 min at room temperature, after which time, 5 µl of vDiD (from 1 mM stock in DMSO) was added followed by an additional incubation for 90 min in the dark at room temperature with mild agitation. The labeled viruses were purified through a NAP-5 gel filtration column (GE Healthcare) in 145 mM NaCl solution buffered with 50 mM HEPES, pH 7.4. Approximately 50% of AF488-labeled particles incorporated detectable amounts of vDiD with minimal contamination by free dye aggregates.

The infectious IAV titer was determined in MDCK or A549 cells after incubation with serially diluted inoculum for 15 h at 37°C. Cells were fixed, permeabilized, blocked and incubated with rabbit R2376 anti-WSN HA antibody (a gift from Dr. D. Steinhauer, Emory University) for 2 h at room temperature. Cells were then washed and incubated with secondary Cy5-conjugated goat anti-rabbit antibodies (Jackson ImmunoResearch, PA) in 10% FBS-containing buffer supplemented with 10 µg/ml Hoechst-33342 for 1 h. The number of infected cells per image field was determined by fluorescence microscopy and normalized to the total number of cells (stained nuclei). The infectious titer (IU/ml) was calculated by taking into account the ratio of the area of well and the image area and correcting for dilution and volume of viral inoculum.

### Virus-cell fusion assay

The β-lactamase (BlaM) assay for virus-cell fusion was carried out as described previously ([24] and Methods S1). Briefly, pseudoviruses bearing β-lactamase-Vpr chimera (BlaM-Vpr) were bound to target cells by centrifugation at 4°C for 30 min at 1550×*g*. Unbound viruses were removed by washing, and fusion was initiated by shifting to 37°C for 90 min, after which time cells were placed on ice and loaded with the CCF4-AM substrate (Life Technologies). The cytoplasmic BlaM activity (ratio of blue to green fluorescence) was measured after an overnight incubation at 12°C, using the Synergy HT fluorescence microplate reader (Bio-Tek, Germany).

### Sub-cellular distribution of IAV, IFITM3 and cholesterol

IAV was pre-bound to A549-IFITM3 cells in the cold, followed by incubation at 37°C for 90 min and immunostaining with mouse anti-IAV-NP (Millipore, Billerica, MA) (when applicable) and rabbit anti-IFITM3 antibody (N-terminus, Abgent, San Diego, CA), as described in [13]. Rat anti-mouse-IgG-FITC (eBioscience, San Diego, CA) and goat anti-rabbit-Cy5 antibodies were used for secondary staining. Cellular distribution of cholesterol was examined by incubation with 0.25 mg/ml filipin added during the incubation with secondary antibodies. Images were collected on a LSM 780 laser scanning microscope (Carl Zeiss, Germany) using a 63× oil immersion objective. All staining methods involved fixation with 2% paraformaldehyde, permeabilization with 0.25% Triton-X100, blocking in with 10% FBS and dilution in phosphate buffered saline (with calcium and magnesium), and sequential incubation with primary and secondary antibodies for 2 h and 1 h, respectively.

### NPC1 knockdown and western blotting

To silence the *NPC1* gene, A549 cells were transduced with five shRNAs encoded by pLK0.1 lentiviral vector (Sigma) and selected with puromycin. The samples for Western blotting were processed as described in [24]. The NPC1 protein band was detected with rabbit anti-NPC1 (Abcam, Cambridge, MA) and horseradish peroxidase-conjugated Protein G (Bio-Rad, Hercules, CA), using a chemiluminescence reagent from GE Healthcare.

### Single virus imaging and image analysis

Cells grown on glass-bottom Petri dishes (MatTek, MA) were chilled on ice and washed with cold Hank's balanced salt solution (HBSS). Predetermined amount of viral suspension (MOI∼0.01) was added to the cells and spinoculated at 4°C for 20 min. The cells were then washed twice with cold HBSS and placed on the stage of an LSM 780 confocal microscope. Virus entry was initiated by adding 2.5 ml of pre-warmed imaging buffer and imaged at 37°C using a C-Apo 40×/1.2NA water-immersion objective. Three Z-stacks separated by ∼2 µm were acquired every 7–8 s through the MultiTime macro (Carl Zeiss). To block IAV hemifusion and fusion, experiments where performed in HBSS supplemented with 50 mM HEPES/70 mM NH_4_Cl (pH 7.6) or containing 200 nM of BafA1. The time lapse images were first visually inspected to identify vDiD dequenching or loss of mCherry events. The number of relevant events in each experiment was independently determined by two trained individuals. Particle trajectories and their mean/total fluorescence intensities were obtained using Volocity (PerkinElmer, MA). The onset of lipid mixing and the initial slope of vDiD dequenching were determined by fitting to a pair of straight lines (Fig. S11).

### Endosomal pH measurements

IAV particles were co-labeled with the AF488 dye (pH-insensitive) and CypHer5E, which fluoresces brighter at acidic pH. The ratios of the CypHer5E and AF488 signals were converted to pH values using a calibration curve obtained by exposing coverslip-immobilized viruses to citrate-phosphate buffers of different acidity (Fig. S7). Images were collected from 3 different fields, and sum of single-particle fluorescence was calculated. The mean ratios of CypHer5E to AF488 signals as a function of pH were used for the calibration curve. Cells were inoculated with labeled viruses for 45 min at 37°C, as described above. Images were collected from at least 10 different fields, and single particle-based ratio of fluorescence signals was calculated. Outliers with a near-background CypHer5E signal were rejected to reduce the uncertainty in pH measurements.

### Statistical analyses

Statistical significance was assessed using the pairwise t-test or rank sum test. Single-particle fusion events in control and IFITM3 expressing cells were compared by the z-test.

## Supporting Information

Figure S1 **Characterization of AlexaFluor488 and vDiD co-labeled IAV.** (A) vDiD and AF488 co-labeling does not strongly affect IAV infectivity. Mock-labeling of viral particles was carried out by subjecting 100 µg of H1N1 A/PR/8/34 virus preparation to the same solvents/buffer, incubation periods and purification protocol as that for labeling, but in the absence of AF488 and vDiD dyes. Infectious titer was estimated, as described in Materials and Methods. Error bars are standard deviations (n = 10). (B, C) Immunostaining of AF488-labeled H1N1 A/PR/8/34 virions (B) and of ASLV Env-pseudotyped retroviral particles (C, negative control) with anti-HA antibody (red).
(PDF)

Figure S2 **Effect of oleic acid (OA) on IAVpp fusion with A549 and A549-IFITM3 cells.** BlaM-Vpr carrying pseudoviruses (MOI = 1) were bound to cells in the cold. Unbound virus was washed out, and the samples were treated with either 100 µM OA, 70 mM NH_4_Cl or left untreated. Fusion was allowed to proceed by shifting to 37°C for 90 min. Data are means and SEM for 2 triplicate experiments. NS, not significant.
(PDF)

Figure S3 **Examples of fast vDiD dequenching events in A549 and MDCK cells.** Relatively quick vDiD (red) dequenching events obtained by single particle tracking are shown for A549, A549-IFITM3, MDCK and MDCK-IFITM3 cells. The AF488 signal is shown in green and the ratio of vDiD and AF488 signals is shown in blue. Arrows mark sudden increases in the vDiD signal. a.u., arbitrary units.
(PDF)

Figure S4 **Correlation between the lag time before lipid mixing and the rate of vDiD dequenching (A) and the initial rates of vDiD dequenching (B).** (A) The time of commencement of hemifusion (T_H_) and the initial rate of dequenching was determined as described in Materials and Methods. These parameters are uncorrelated (R^2^<0.19 for all). (B) The initial rates of vDiD dequenching were determined for A549-Vector, A549-IFITM3, MDCK and CHO cells. Error bars are SEM from >20 tracks. *, P<0.02.
(PDF)

Figure S5 **Relationship between IAV lipid mixing activity and infection.** The fraction of A549 cells where at least one lipid mixing event was observed within 1 h at 37°C, and the fraction of cells that became infected within 15 h at 37°C were estimated as described in Methods S1. Infectivity data were collected from 5 image fields each, with >30 cells per field. Particle-to infectivity ratio was calculated from the fraction of infected cells and the average number of virions bound to cells. Live cell imaging experiments (n = 10 for A549 and n = 6 for MDCK cells) yielded the number of cells receiving at least hemifusion event.
(PDF)

Figure S6 **Subcellular distribution of cholesterol and levels of total and free cellular cholesterol.** (A) Total cellular filipin was estimated by calculating the filipin fluorescence intensity over the entire image field (after subtracting the background signal) and normalizing by the number of cells per field. Data are means and standard deviations for 4 and 6 fields for A549 and A549-IFITM3 cells (131 and 184 cells), respectively. (B, C) Total and free cellular cholesterol (in µg/10^6^ cells) were measured by a fluorimetric enzymatic assay using the Cholesterol Kit from Sigma-Aldrich. Data are means and standard deviations from 2 measurements performed with duplicate samples. ***, P<0.001; *, P<0.03.
(PDF)

Figure S7 **Calibration of labeled IAV as a pH-sensor.** AF488- and CypHer5E- labeled IAV particles were attached to poly-L-lysine coated coverslips, and the ratio of two fluorescence signals was measured in citrate-phosphate buffers of different acidity. (A) Top and bottom panels are images of labeled IAV at neutral pH and low pH, respectively. (B) The total signal for each dye was determined after thresholding and the CypHer5E/AF488 ratio at different pH are plotted. Error bars are standard deviations for 3 different imaged fields for each pH value. The line indicates a first order polynomial fit to the data, which served as a pH calibration curve.
(PDF)

Figure S8 **An example of single IAV lipid mixing event in CHO cells.** (A) Image panels show entry of an AF488 (green) and vDiD (red) labeled virus into a CHO cell that culminates in vDiD dequenching (arrow). (B) Fluorescence intensity profiles of AF488 and vDiD obtained by tracking the virion shown in panel A.
(PDF)

Figure S9 **pH distribution in IAV carrying endosomes of CHO cells.** Shown are the distributions of endosomal pH in CHO cells pretreated with 40 µM of U18666A for 12 h or left untreated. Cells were incubated with AF488/Cypher5E-labeled IAV, and endosomal pH was measured as described in Materials and Methods. U18666A increased endosomal acidity (P<0.001).
(PDF)

Figure S10 **Incoming IAV tends to colocalize with IFITM3-positive endosomes.** A549-IFITM3 cells were allowed to internalize IAV for 90 min at 37°C and immunostained for the IAV-NP using mouse antibody (Millipore, Billerica, MA) and for IFITM3. The enlarged boxed area is shown on the right. IAV and IFITM3 puncta were identified by thresholding and object identification. The extent of colocalization was estimated by counting IAV puncta, which exhibited a volumetric overlap of at least 50% with IFITM3 puncta, and normalizing over all IAV puncta. The number in the right corner is the mean % colocalization and standard deviation for 7 image fields.
(PDF)

Figure S11 **A line-fitting approach to determining the onset and the initial rate of vDiD dequenching in single IAV fusion experiments.** Fitting the vDiD dequenching traces with two straight lines yields the time of hemifusion (T_H_) and the initial slope of dequenching.
(PDF)

Methods S1 **Description of additional methods employed in this study.**
(DOCX)

Movie S1 **Lipid mixing between single vDiD-labeled IAV and an endosome in A549 cells.** IAV co-labeled with AF488 (green) and vDiD (red) was incubated with A549 cells at 37°C. The lipid mixing event (hemifusion) is manifested in marked increase of vDiD fluorescence. The numbers in the upper right corner show time after raising the temperature (min∶sec∶msec). Scale bar is 10 µm. For details, see Fig. 2A, B.
(AVI)

Movie S2 **Lipid mixing between single vDiD-labeled IAV and an endosome in A549-IFITM3 cell.** IAV co-labeled with AF488 (green) and vDiD (red) was incubated with cells at 37°C. The lipid mixing event (hemifusion) is manifested in marked increase of vDiD fluorescence. The numbers in the upper right corner show time after raising the temperature (min∶sec∶msec). For details, see Fig. 2D, E.
(AVI)

Movie S3 **Lipid mixing upon entry of single vDiD-labeled IAV into an MDCK-IFITM3 cell.** IAV co-labeled with AF488 (green) and vDiD (red) was incubated with cells at 37°C. Lipid mixing (hemifusion) is seen as marked increase in the vDiD signal. The numbers in the upper right corner show time after raising the temperature (min∶sec∶msec). For details, see Fig. 2F, G.
(AVI)

Movie S4 **IAVpp fusion with an endosome in A549 cell.** A single IAV pseudovirus co-labeled with YFP-Vpr (green) and Gag-iCherry (red) releases its content marker (iCherry) after entering the cell. The numbers in the upper right corner show time after raising the temperature (min∶sec∶msec). For details, see Fig. 4A, B.
(AVI)

Movie S5 **IAVpp fusion with an MDCK cell.** A single IAV pseudovirus co-labeled with YFP-Vpr (green) and Gag-iCherry (red) releases its content marker (iCherry) after entering the cell. The numbers in the upper right corner show time after raising the temperature (min∶sec∶msec). For details, see Fig. 4C, D.
(AVI)
