# Supplementary figures and images for: IFITM3 Restricts Influenza A Virus Entry by Blocking the Formation of Fusion Pores following Virus-Endosome Hemifusion
Source: PLoS Pathog. 2014 Apr 3;10(4):e1004048. doi: 10.1371/journal.ppat.1004048 (PMC3974867; doi:10.1371/journal.ppat.1004048)

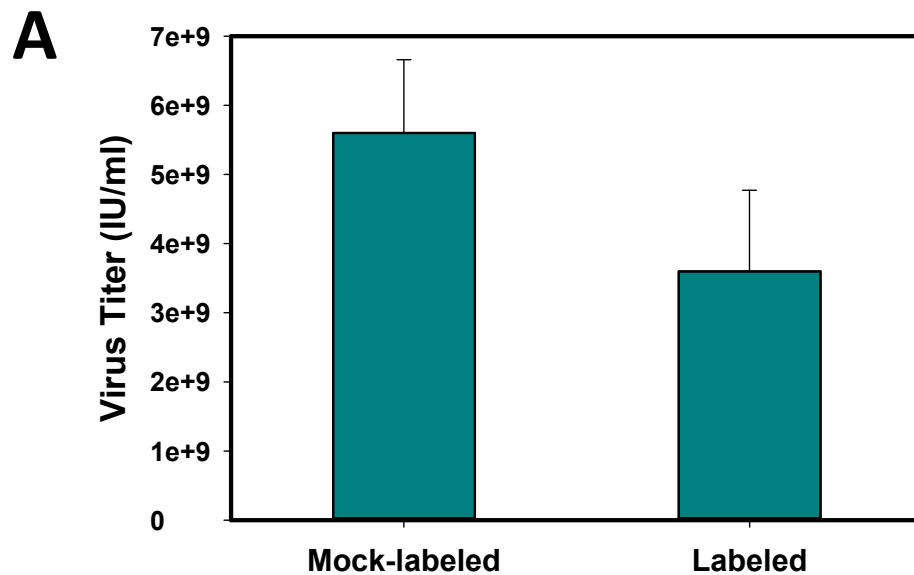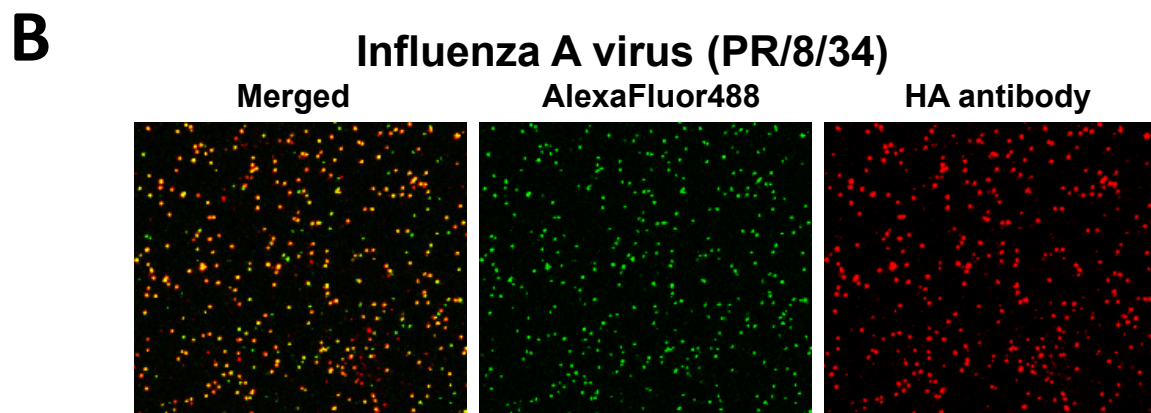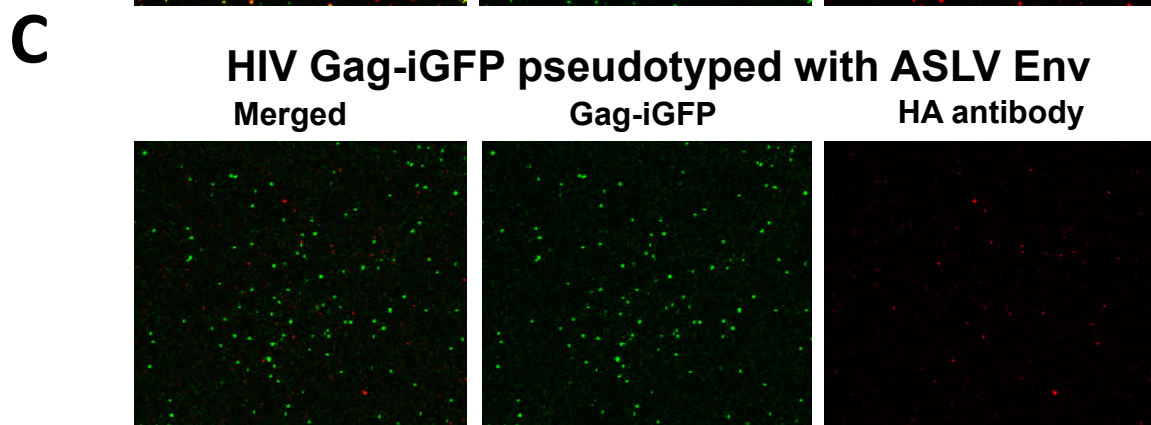

Figure S1

Supplement: Figure S1 — Characterization of AlexaFluor488 and vDiD co-labeled IAV. (A) vDiD and AF488 co-labeling does not strongly affect IAV infectivity. Mock-labeling of viral particles was carried out by subjecting 100 µg of H1N1 A/PR/8/34 virus preparation to the same solvents/buffer, incubation periods and purification protocol as that for labeling, but in the absence of AF488 and vDiD dyes. Infectious titer was estimated, as described in Materials and Methods. Error bars are standard deviations (n = 10). (B, C) Immunostaining of AF488-labeled H1N1 A/PR/8/34 virions (B) and of ASLV Env-pseudotyped retroviral particles (C, negative control) with anti-HA antibody (red). (PDF) [file ppat.1004048.s001.pdf]

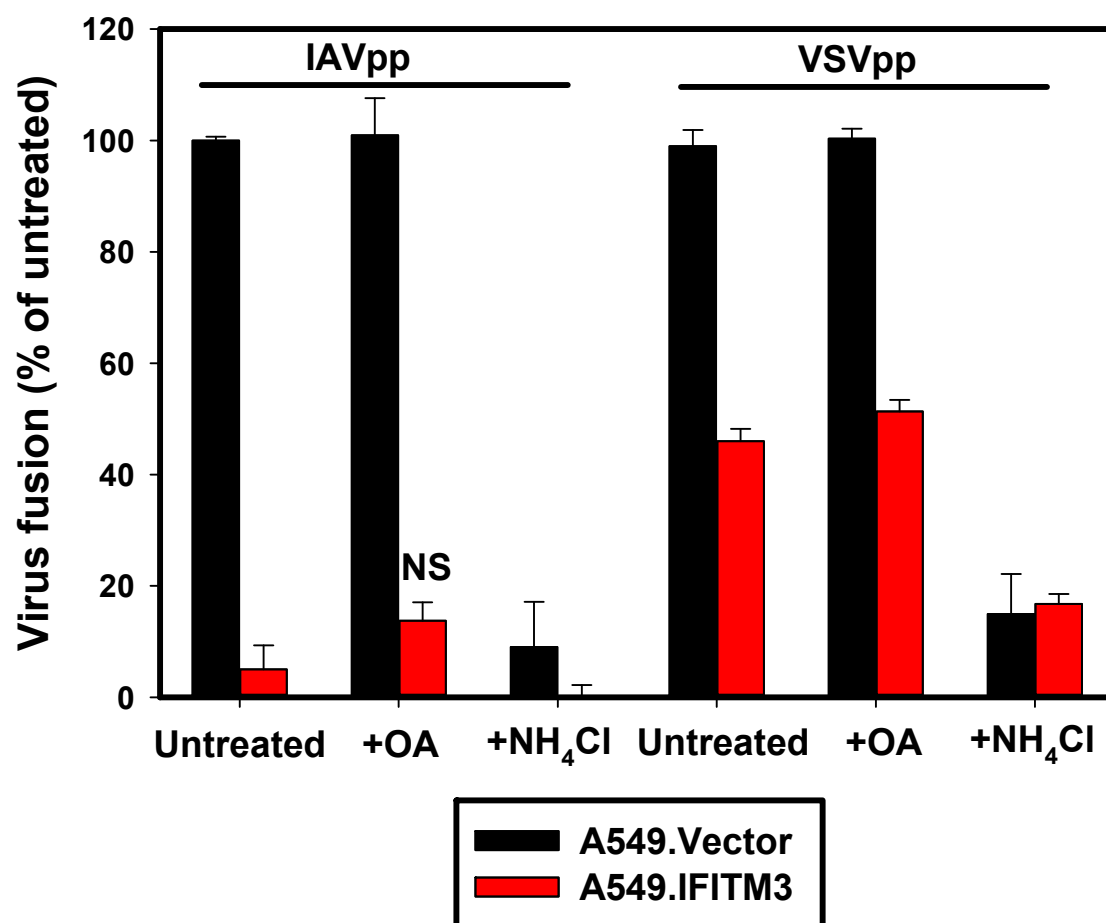

Figure S2

Supplement: Figure S2 — Effect of oleic acid (OA) on IAVpp fusion with A549 and A549-IFITM3 cells. BlaM-Vpr carrying pseudoviruses (MOI = 1) were bound to cells in the cold. Unbound virus was washed out, and the samples were treated with either 100 µM OA, 70 mM NH4Cl or left untreated. Fusion was allowed to proceed by shifting to 37°C for 90 min. Data are means and SEM for 2 triplicate experiments. NS, not significant. (PDF) [file ppat.1004048.s002.pdf]

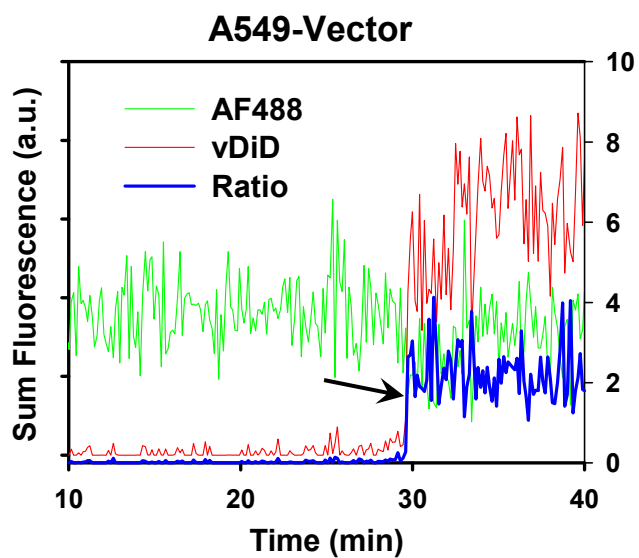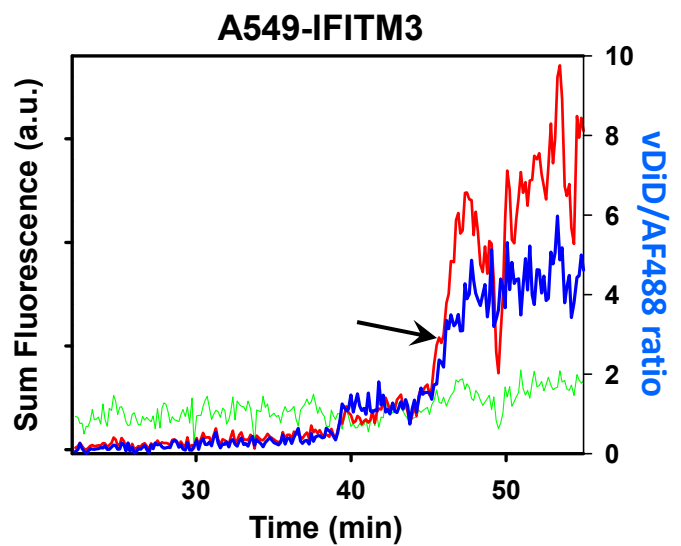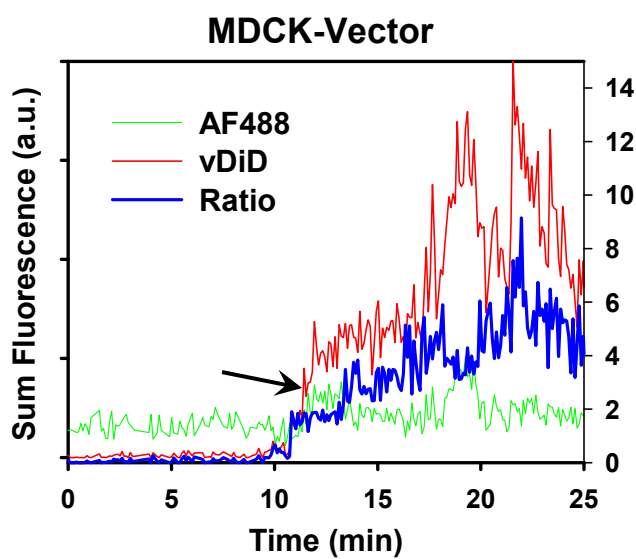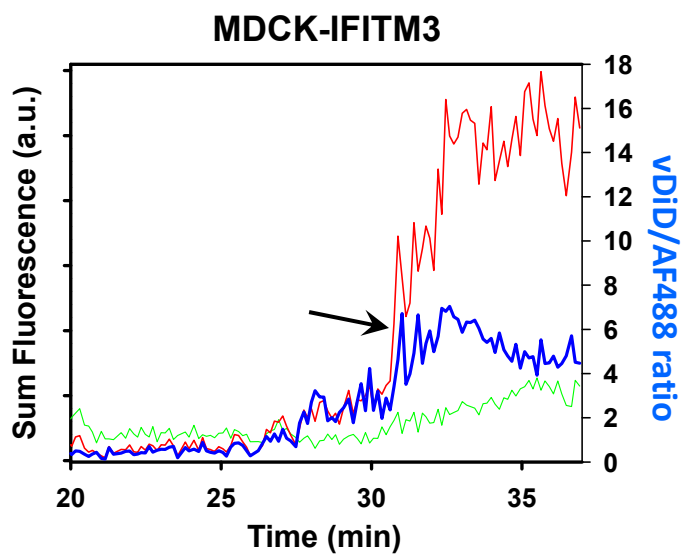

Figure S3

Supplement: Figure S3 — Examples of fast vDiD dequenching events in A549 and MDCK cells. Relatively quick vDiD (red) dequenching events obtained by single particle tracking are shown for A549, A549-IFITM3, MDCK and MDCK-IFITM3 cells. The AF488 signal is shown in green and the ratio of vDiD and AF488 signals is shown in blue. Arrows mark sudden increases in the vDiD signal. a.u., arbitrary units. (PDF) [file ppat.1004048.s003.pdf]

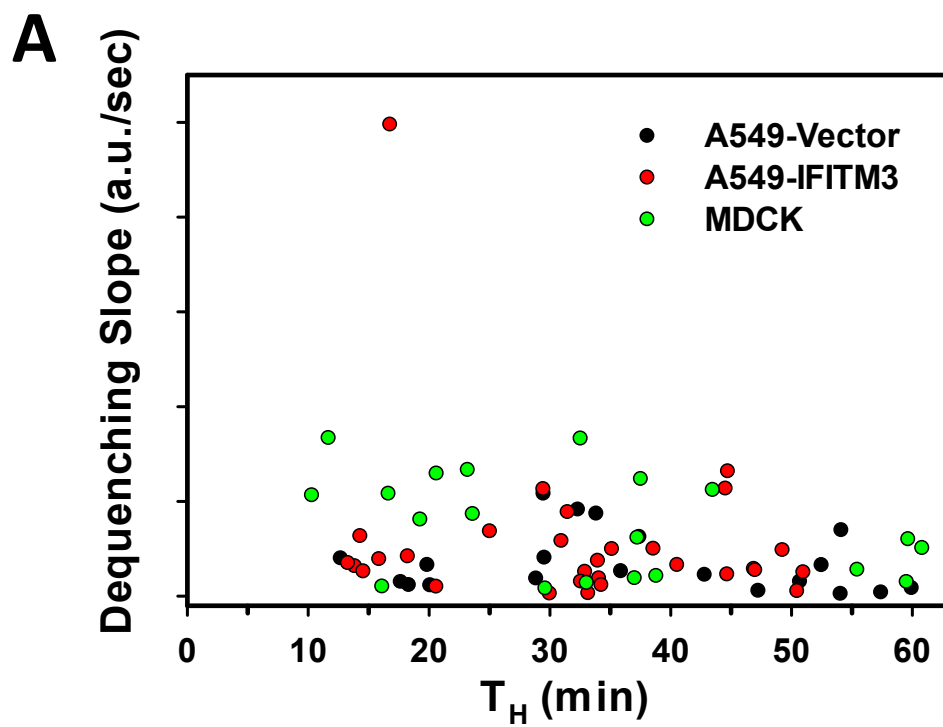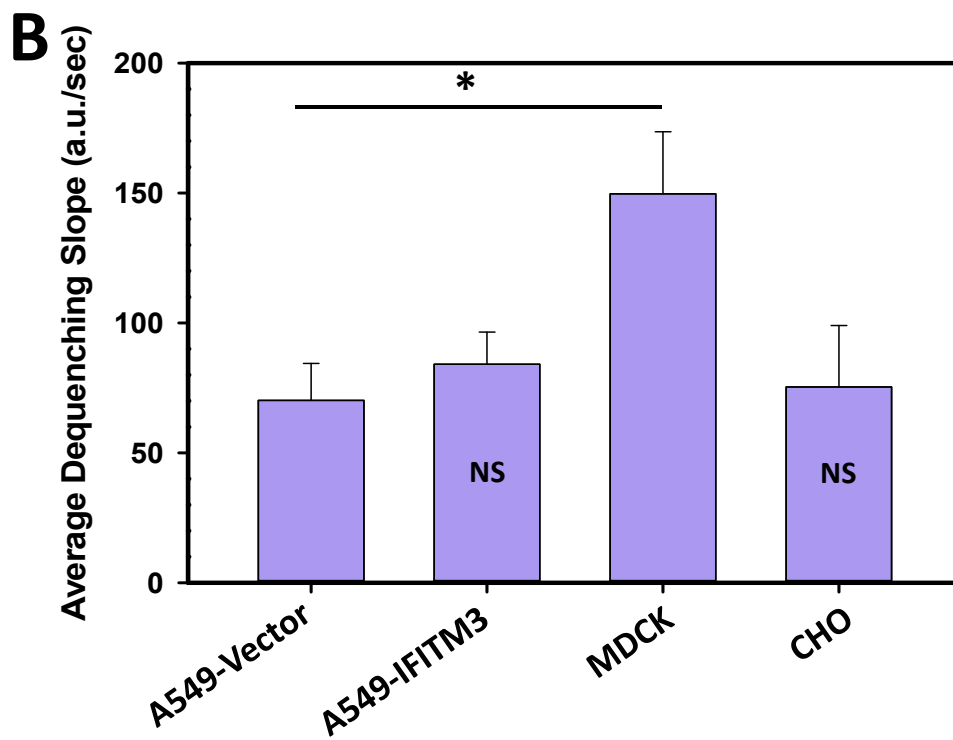

Figure S4

Supplement: Figure S4 — Correlation between the lag time before lipid mixing and the rate of vDiD dequenching (A) and the initial rates of vDiD dequenching (B). (A) The time of commencement of hemifusion (TH) and the initial rate of dequenching was determined as described in Materials and Methods. These parameters are uncorrelated (R2<0.19 for all). (B) The initial rates of vDiD dequenching were determined for A549-Vector, A549-IFITM3, MDCK and CHO cells. Error bars are SEM from >20 tracks. *, P<0.02. (PDF) [file ppat.1004048.s004.pdf]

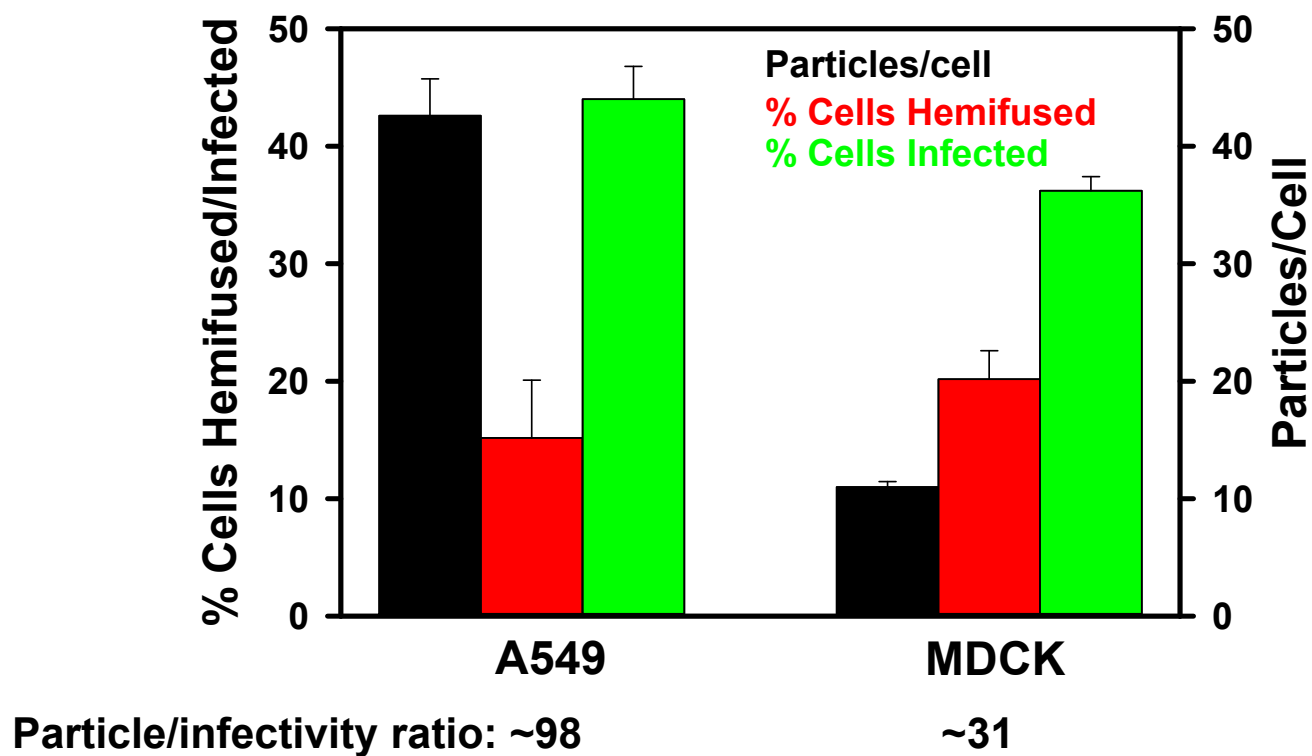

Figure S5

Supplement: Figure S5 — Relationship between IAV lipid mixing activity and infection. The fraction of A549 cells where at least one lipid mixing event was observed within 1 h at 37°C, and the fraction of cells that became infected within 15 h at 37°C were estimated as described in Methods S1. Infectivity data were collected from 5 image fields each, with >30 cells per field. Particle-to infectivity ratio was calculated from the fraction of infected cells and the average number of virions bound to cells. Live cell imaging experiments (n = 10 for A549 and n = 6 for MDCK cells) yielded the number of cells receiving at least hemifusion event. (PDF) [file ppat.1004048.s005.pdf]

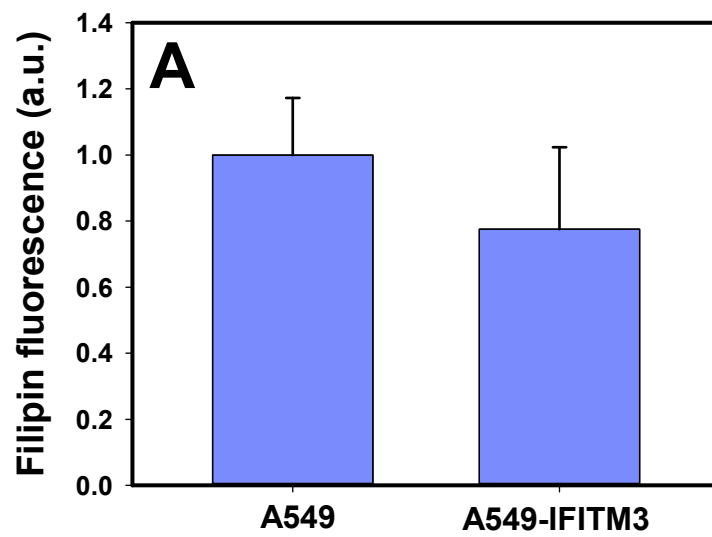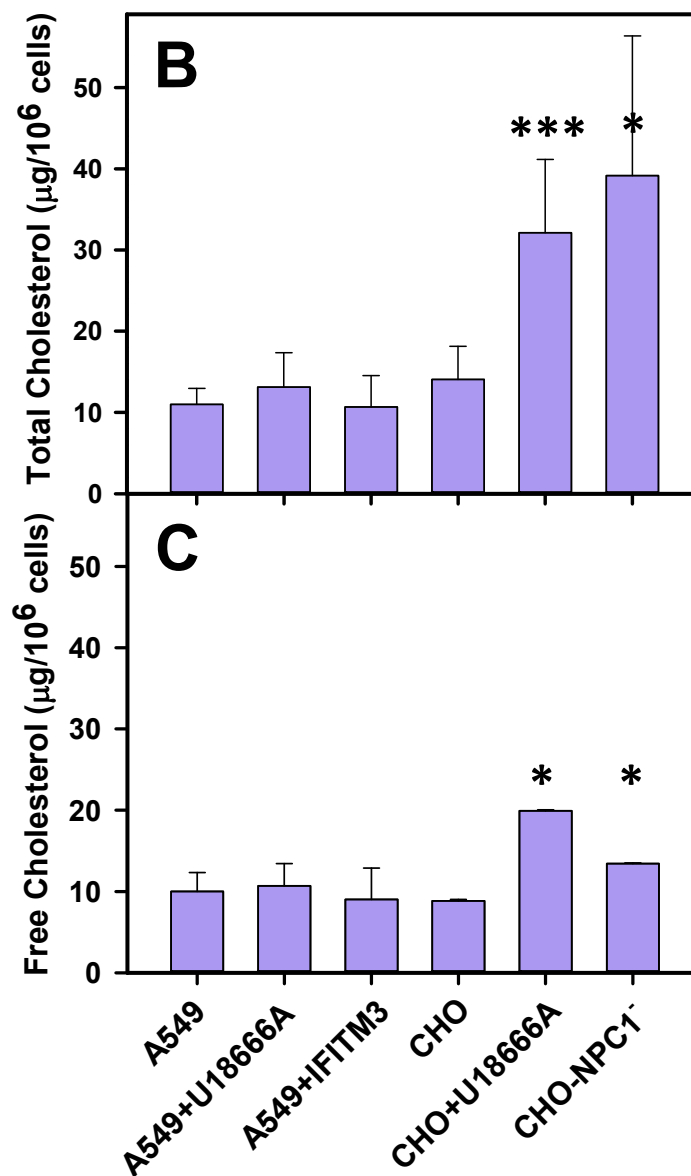

Figure S6

Supplement: Figure S6 — Subcellular distribution of cholesterol and levels of total and free cellular cholesterol. (A) Total cellular filipin was estimated by calculating the filipin fluorescence intensity over the entire image field (after subtracting the background signal) and normalizing by the number of cells per field. Data are means and standard deviations for 4 and 6 fields for A549 and A549-IFITM3 cells (131 and 184 cells), respectively. (B, C) Total and free cellular cholesterol (in µg/106 cells) were measured by a fluorimetric enzymatic assay using the Cholesterol Kit from Sigma-Aldrich. Data are means and standard deviations from 2 measurements performed with duplicate samples. ***, P<0.001; *, P<0.03. (PDF) [file ppat.1004048.s006.pdf]

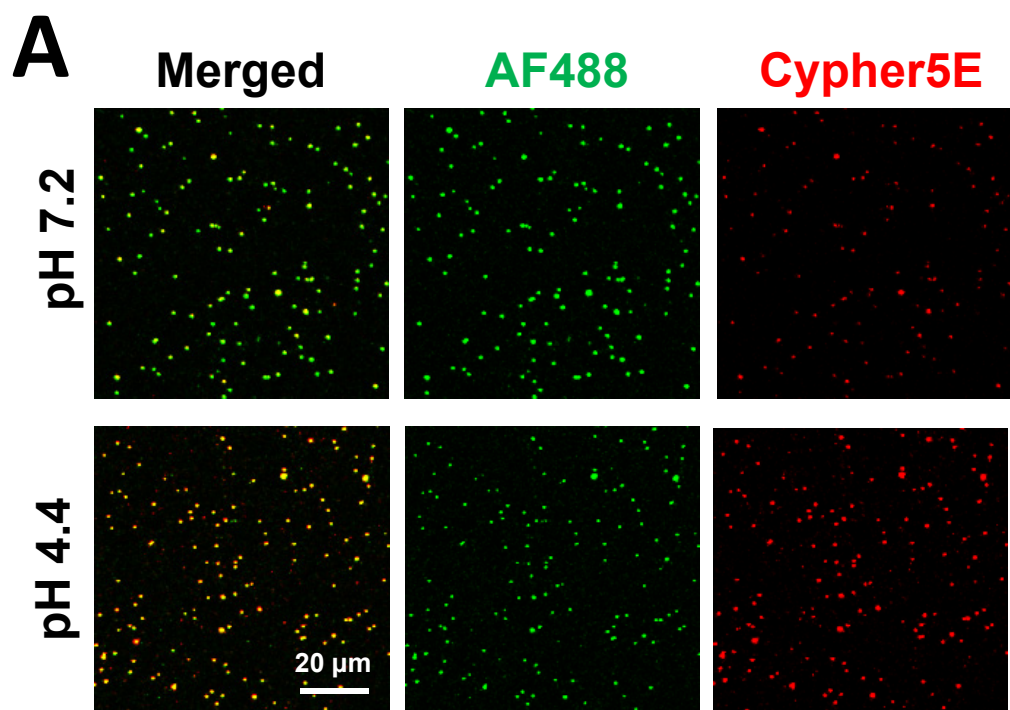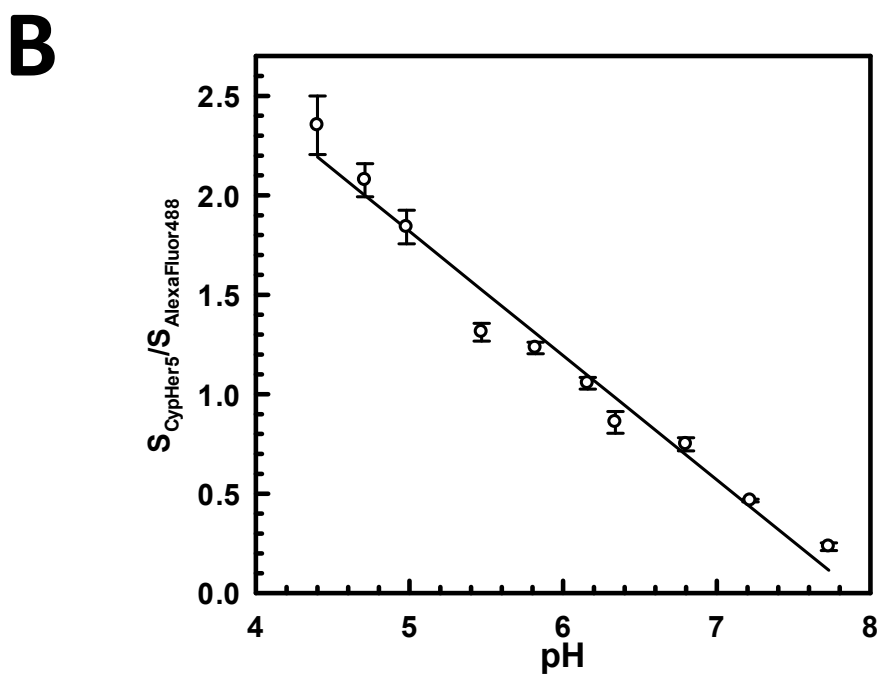

Figure S7

Supplement: Figure S7 — Calibration of labeled IAV as a pH-sensor. AF488- and CypHer5E- labeled IAV particles were attached to poly-L-lysine coated coverslips, and the ratio of two fluorescence signals was measured in citrate-phosphate buffers of different acidity. (A) Top and bottom panels are images of labeled IAV at neutral pH and low pH, respectively. (B) The total signal for each dye was determined after thresholding and the CypHer5E/AF488 ratio at different pH are plotted. Error bars are standard deviations for 3 different imaged fields for each pH value. The line indicates a first order polynomial fit to the data, which served as a pH calibration curve. (PDF) [file ppat.1004048.s007.pdf]

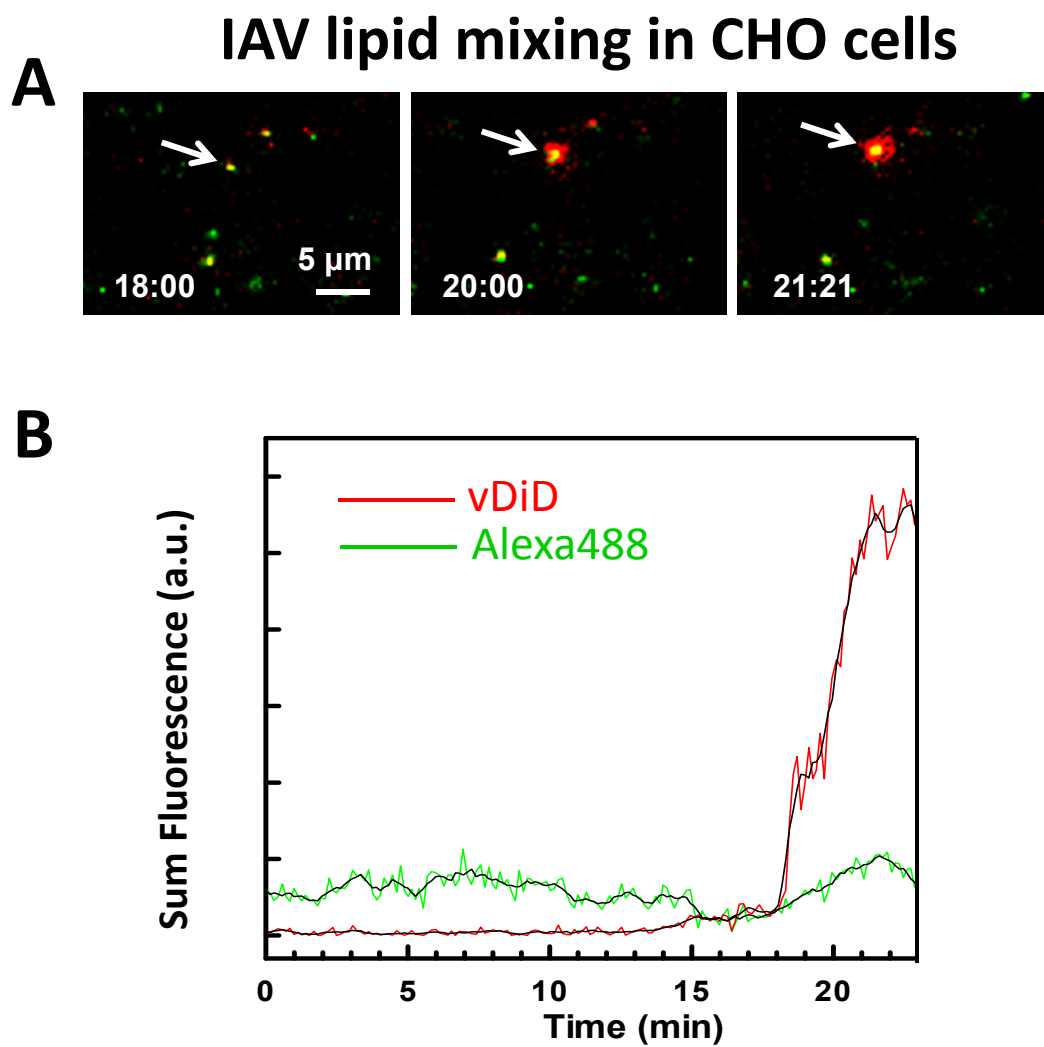

Figure S8

Supplement: Figure S8 — An example of single IAV lipid mixing event in CHO cells. (A) Image panels show entry of an AF488 (green) and vDiD (red) labeled virus into a CHO cell that culminates in vDiD dequenching (arrow). (B) Fluorescence intensity profiles of AF488 and vDiD obtained by tracking the virion shown in panel A. (PDF) [file ppat.1004048.s008.pdf]

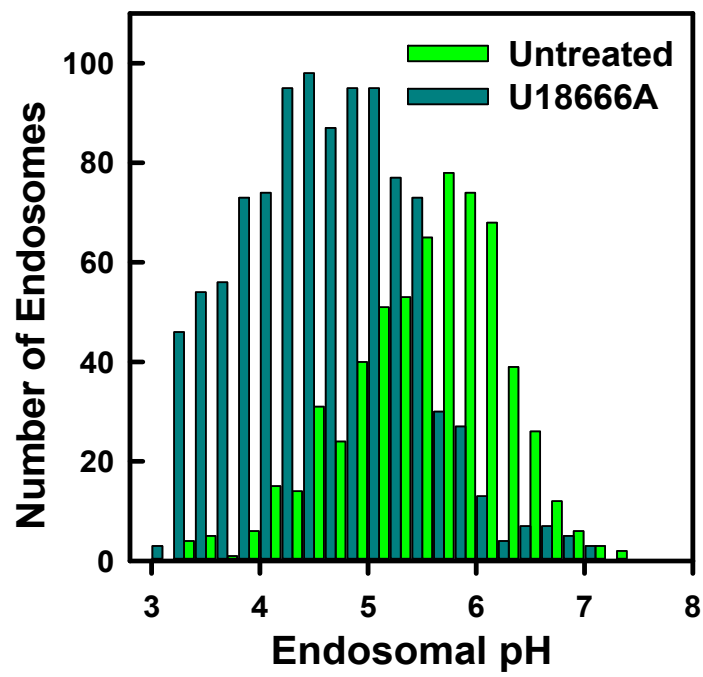

Figure S9

Supplement: Figure S9 — pH distribution in IAV carrying endosomes of CHO cells. Shown are the distributions of endosomal pH in CHO cells pretreated with 40 µM of U18666A for 12 h or left untreated. Cells were incubated with AF488/Cypher5E-labeled IAV, and endosomal pH was measured as described in Materials and Methods. U18666A increased endosomal acidity (P<0.001). (PDF) [file ppat.1004048.s009.pdf]

### A549-IFITM3 Cells

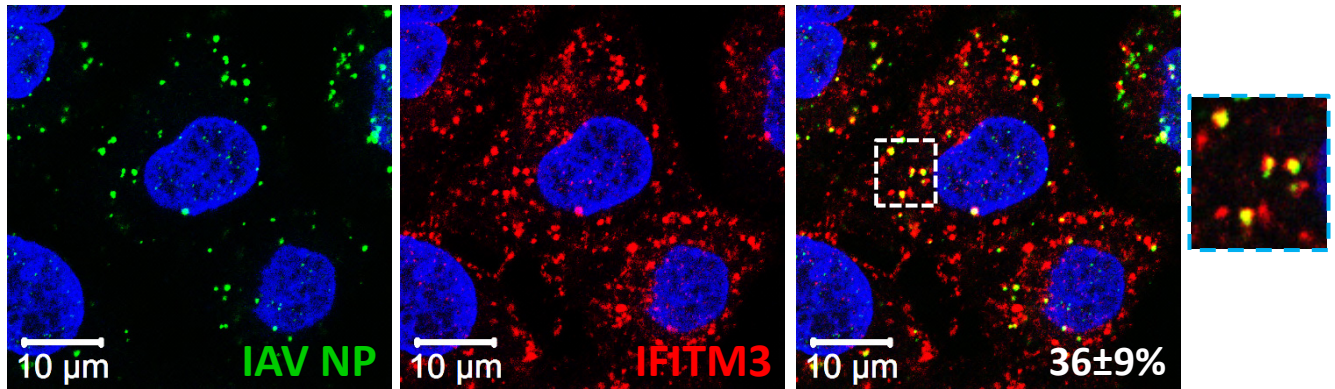

Figure S10

Supplement: Figure S10 — Incoming IAV tends to colocalize with IFITM3-positive endosomes. A549-IFITM3 cells were allowed to internalize IAV for 90 min at 37°C and immunostained for the IAV-NP using mouse antibody (Millipore, Billerica, MA) and for IFITM3. The enlarged boxed area is shown on the right. IAV and IFITM3 puncta were identified by thresholding and object identification. The extent of colocalization was estimated by counting IAV puncta, which exhibited a volumetric overlap of at least 50% with IFITM3 puncta, and normalizing over all IAV puncta. The number in the right corner is the mean % colocalization and standard deviation for 7 image fields. (PDF) [file ppat.1004048.s010.pdf]

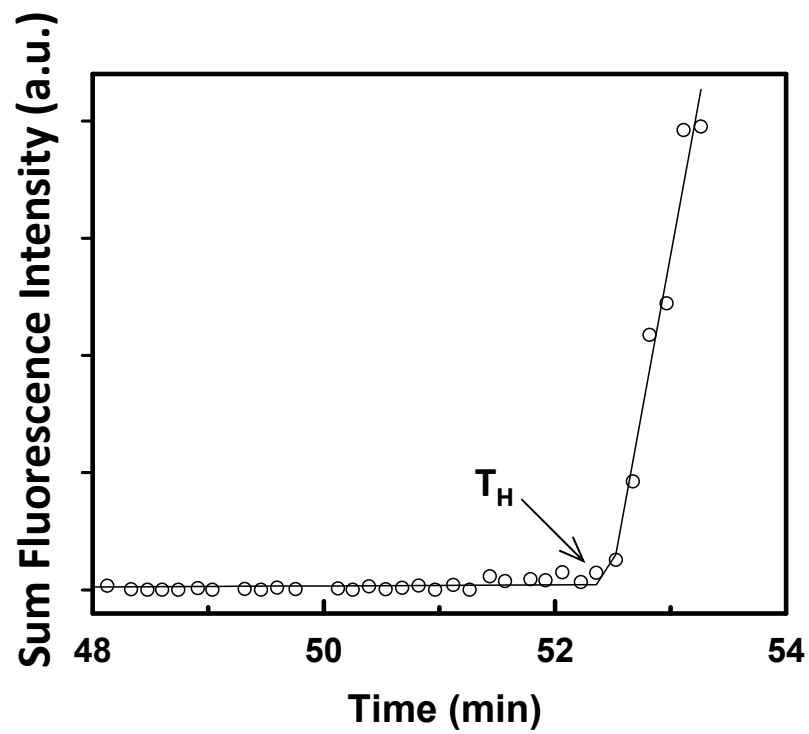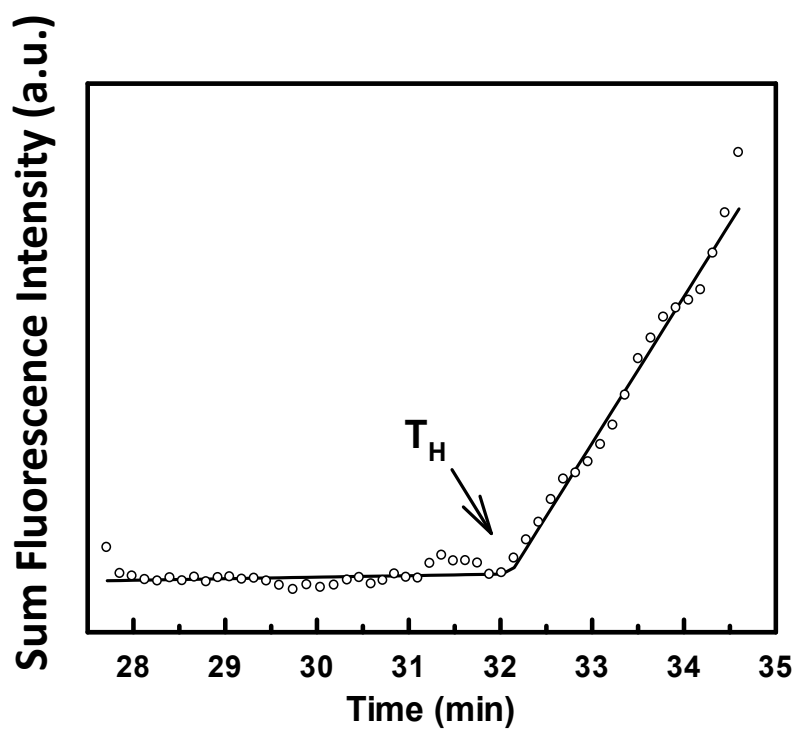

Figure S11

Supplement: Figure S11 — A line-fitting approach to determining the onset and the initial rate of vDiD dequenching in single IAV fusion experiments. Fitting the vDiD dequenching traces with two straight lines yields the time of hemifusion (TH) and the initial slope of dequenching. (PDF) [file ppat.1004048.s011.pdf]
