# Supplementary material for: IFITM3 Restricts Influenza A Virus Entry by Blocking the Formation of Fusion Pores following Virus-Endosome Hemifusion
Source: PLoS Pathog. 2014 Apr 3;10(4):e1004048. doi: 10.1371/journal.ppat.1004048 (PMC3974867; doi:10.1371/journal.ppat.1004048)
Supplement: Methods S1 — Description of additional methods employed in this study. (DOCX) [file ppat.1004048.s012.docx]

**Methods S1**

**Single virus immunostaining for HA.** AF488-labeled H1N1 A/PR/8/34 at an appropriate dilution in PBS was bound to poly-lysine-coated 8-chambered cover slips (Nunc Lab-Tek, Rochester, NY) for 30 min at 4ºC. After removing unbound viruses by washing, viruses were immunostained with rabbit R2376 anti-WSN HA for 2 hours at room-temperature, washed and incubated with goat anti-rabbit Cy5-conjugated IgG for 1 hour. Immunostaining of HIV-Gag-iGFP pseudotyped with Avian Sarcoma and Leukosis Virus envelope glycoprotein (ASLV-EnvA) with the same antibody served as the negative control.

**Relationship between particle infectivity and lipid mixing activity.** AF488- and vDiD-labeled IAV particles were pre-bound to cells in the cold, and 5 image fields per condition were collected to determine the number of bound particles per cell prior to initiation of entry. In order to mimic conditions of live cell imaging, entry was initiated by the addition of warm imaging buffer followed by incubation for 1 hour at 37ºC. At this point, the buffer was replaced with growth medium supplemented with 10% FBS, and cells were further incubated for 14 h prior to assessing the number of infected cells per image field by immunostaining for IAV HA, as described above. The fraction of cells hosting hemifusion and the fraction of infecting cells was calculated based on the number of cells containing at least one vDiD-dequenched double-labeled particle in live cell experiments, and those that stained positive for infectivity, respectively.

**Measurements of the total and free cellular cholesterol.** The overall cholesterol levels were determined by the Cholesterol Kit (Sigma-Aldrich), as per the manufacturer’s protocol. Briefly, cells were grown to ~90% confluency on 60 mm cell culture dishes, washed with PBS and harvested with CellStripper (Mediatech). Cells were then centrifuged, washed and re-suspended in PBS. Cells were counted, and cellular cholesterol was extracted with choloform-isopropanol-IGEPAL630-CA with sonication. Cellular debris was cleared by centrifugation, and the supernatant was air-dried at 50^o^C for 20 min to evaporate solvents. Dry lipids were re-suspended in 200 μl of the kit assay buffer. The kit measures free cholesterol, whereas total cholesterol content (which includes the esterified component) was detected by application of cholesterol esterase per the manufacturer’s protocol.
